# A H2AX–CARP-1 Interaction Regulates Apoptosis Signaling Following DNA Damage

**DOI:** 10.3390/cancers11020221

**Published:** 2019-02-14

**Authors:** Sreeja C. Sekhar, Jaganathan Venkatesh, Vino T. Cheriyan, Magesh Muthu, Edi Levi, Hadeel Assad, Paul Meister, Vishnu V. Undyala, James W. Gauld, Arun K. Rishi

**Affiliations:** 1 John D. Dingell Veterans Administration Medical Center, Detroit, MI 48201, USA; sreejacsekhar@gmail.com (S.C.S.); jaganvibt@gmail.com (J.V.); vinorcc@gmail.com (V.T.C.); magesh.research@gmail.com (M.M.); Edi.Levi@va.gov (E.L.); 2 Department of Oncology, Karmanos Cancer Institute, Detroit, MI 48201, USA; Assadh@karmanos.org; 3 Department of Chemistry and Biochemistry, University of Windsor, Windsor, ON N9B 3P4, Canada; meisterp@uwindsor.ca (P.M.); gauld@uwindsor.ca (J.W.G.); 4 Cardiovascular Research Institute, School of Medicine, Wayne State University, Detroit, MI 48201, USA; uvreddi@gmail.com; 5 Molecular Therapeutics Program, Barbara Ann Karmanos Cancer Institute, Detroit, MI 48201, USA

**Keywords:** CCAR1/CARP-1, γH2AX, apoptosis, chemotherapeutics, cancer cells

## Abstract

Cell Cycle and Apoptosis Regulatory Protein (CARP-1/CCAR1) is a peri-nuclear phosphoprotein that regulates apoptosis via chemotherapeutic Adriamycin (doxorubicin) and a novel class of CARP-1 functional mimetic (CFM) compounds. Although Adriamycin causes DNA damage, data from Comet assays revealed that CFM-4.16 also induced DNA damage. Phosphorylation of histone 2AX (γH2AX) protein is involved in regulating DNA damage repair and apoptosis signaling. Adriamycin or CFM-4.16 treatments inhibited cell growth and caused elevated CARP-1 and γH2AX in human breast (HBC) and cervical cancer (HeLa) cells. In fact, a robust nuclear or peri-nuclear co-localization of CARP-1 and γH2AX occurred in cells undergoing apoptosis. Knock-down of CARP-1 diminished γH2AX, their co-localization, and apoptosis in CFM-4.16- or Adriamycin-treated cells. We found that CARP-1 directly binds with H2AX, and H2AX interacted with CARP-1, but not CARP-1 (Δ600–652) mutant. Moreover, cells expressing CARP-1 (Δ600–652) mutant were resistant to apoptosis, and had diminished levels of γH2AX, when compared with cells expressing wild-type CARP-1. Mutagenesis studies revealed that H2AX residues 1–35 harbored a CARP-1-binding epitope, while CARP-1 amino acids 636–650 contained an H2AX-interacting epitope. Surface plasmon resonance studies revealed that CARP-1 (636–650) peptide bound with H2AX (1–35) peptide with a dissociation constant (K_d_) of 127 nM. Cells expressing enhanced GFP (EGFP)-tagged H2AX (1–35) peptide or EGFP-tagged CARP-1 (636–650) peptide were resistant to inhibition by Adriamycin or CFM-4.16. Treatment of cells with transactivator of transcription (TAT)-tagged CARP-1 (636–650) peptide resulted in a moderate, statistically significant abrogation of Adriamycin-induced growth inhibition of cancer cells. Our studies provide evidence for requirement of CARP-1 interaction with H2AX in apoptosis signaling by Adriamycin and CFM compounds.

## 1. Introduction

Apoptosis is an important physiological process that is often utilized during the development and maintenance of normal tissue homeostasis in organisms. Defects in the pathways that control apoptosis are implicated as causal or contributing factors in degenerative disorders, auto-immune diseases, and cancer [1,2]. To date, genetic and biochemical studies identified two major pathways of apoptosis and a host of biochemical components that function to regulate these pathways in a variety of cell and organism models [2,3]. A number of extrinsic or intrinsic regulators of apoptosis were and continue to be exploited to design novel therapeutic strategies [4]. The regulators of apoptosis signaling, including the ones that are yet to be discovered, therefore, represent attractive avenues for development of intervention strategies for such pathologies where apoptosis is defective [3,4]. In this regard, elucidation of novel mechanism(s) of apoptosis signaling would be useful in further defining cellular apoptotic pathways.

Cell Cycle and Apoptosis Regulatory Protein (CARP-1/CCAR1) is a peri-nuclear phospho-protein that regulates cell growth and apoptosis signaling [5,6]. CARP-1 functions as a co-activator of growth signaling via steroid thyroid nuclear receptors and oncogenic signaling via β-catenin, and co-activates the glucocorticoid receptor for adipocyte differentiation, the anaphase-promoting complex cyclosome (APC/C) E3 ligase, and tumor suppressor p53 for cell-cycle control and apoptosis [7,8,9,10]. High-throughput screening of a chemical library yielded multiple, small-molecule inhibitors (SMIs) of CARP-1 interaction with the APC2 component of APC/C E3 ligase. These novel compounds, termed CARP-1 functional mimetics (CFMs), bind with CARP-1, stimulate CARP-1 levels, and inhibit cell growth in part by inducing cell-cycle arrest and apoptosis [11,12,13,14,15,16,17,18]. CARP-1 tyrosine (Y)192 was previously found to regulate apoptosis signaling following pharmacologic inhibition of epidermal growth factor receptors (EGFR), while CARP-1-dependent apoptosis involved activation of stress-activated protein kinase (SAPK) p38α/β, and caspases-8 and -9 [6,10]. Depletion of CARP-1 on the other hand confers resistance to apoptosis induced by CFMs, as well as the chemotherapeutics Adriamycin (doxorubicin), etoposide, and EGFR tyrosine kinase inhibitors (TKIs).

Adriamycin was proposed to exert its cytotoxic effect through a number of mechanisms, including (a) inhibition of topoisomerase II, resulting in the formation of DNA double-strand breaks, and (b) formation of intercalating Adriamycin DNA adducts that prevent DNA replication. Overall, DNA damage is key to Adriamycin-induced cell death. Prior studies reported that phosphorylation of histone 2AX (H2AX) at serine 139 (γ-H2AX) is involved in DNA damage repair and apoptotic signaling by Ataxia–telangiectasia mutated (ATM)/ ATM and RAD3 related (ATR) and c-jun N-terminal Kinases (JNKs), respectively [19,20,21]. Given the critical role of CARP-1 in apoptotic signaling by Adriamycin and CFM compounds, and the fact that stress-activated kinases like JNKs and phosphoinositide 3-kinase (PI3Ks) are involved in apoptosis signaling by CARP-1 [5,12,13,14,15,16,17,18] and H2AX [19,20,21], we speculated that the H2AX–CARP-1 axis could be a key for transduction of apoptotic signaling. Our study reveals that Adriamycin or CFMs promote increase of CARP-1 and γ-H2AX proteins and their co-localization. Furthermore, mutagenesis studies revealed that a direct interaction of CARP-1 with H2AX is necessary for apoptosis.

## 2. Materials and Methods

Cell culture media Dulbecco Modified Eagle Medium (DMEM), Roswell Park Memorial Institute medium (RPMI)-1640, and the antibiotics (streptomycin and penicillin) were purchased from Invitrogen Co. (Carlsbad, CA, USA). Ham’s F-12 medium was purchased from Life Technologies, Inc. (Grand Island, NY, USA), and fetal bovine serum (FBS) was obtained from Denville Scientific Inc. (Metuchen, NJ, USA). The JNK1/2 inhibitor JNK-IN-8 was obtained from Selleck Chemicals, (Houston, TX, USA). The CFM-4 compound was obtained from ChemDiv (San Diego, CA, USA). Structure and synthesis of CFM-4 analog CFM-4.16 was previously described [16]. Clinical-grade Adriamycin (ADR) was obtained from the Harper Hospital Pharmacy, Wayne State University, Detroit, MI, USA. Dimethyl sulfoxide (DMSO), 2-deoxy-glucose, 3-4,5-dimethyltiazol-2-yl-2.5-diphenyl-tetrazolium bromide (MTT), anti-FLAG tag, and anti-actin antibodies were purchased from Sigma Chemical Co (St. Louis, MO, USA). The affinity purified, anti-CARP-1 (α1 and α2) polyclonal antibodies were described previously [5]. Antibodies for Gst-tag, enhanced GFP (EGFP), Myc-tag, phospho, and total H2AX were purchased from Cell Signaling (Beverley, MA, USA).

### 2.1. Cell Lines and Cell Culture

Routine maintenance and culture of MDA-MB-468 (lacking estrogen receptor; ER and having mutant p53) human triple-negative breast cancer (TNBC) cells, human cervical cancer HeLa, human kidney cancer UOK 262, non-small-cell lung cancer H1975, and HCC827 cells was carried out essentially as described previously [5,12,13]. Generation and characterization of MDA-MB-468 cells expressing reduced CARP-1 was described previously [5]. The stable sublines were generated by transfecting the MDA-MB-468 and Hela cells with the pcDNA3 vector, pcDNA3-CARP-1 wild-type (WT)-myc-His clone 6.1.2, pcDNA3-CARP-1(Δ600–650)-myc-His, pcDNA3-EGFP, pcDNA3-EGFP-H2AX (1–35), or pcDNA3-EGFP-CARP-1 (636–650) plasmids, followed by selection in the presence of 800 μg/mL neomycin using described methods [5]. The cell lysates from wild-type, untransfected cells, neomycin-resistant pools, or individual sublines were then subjected to MTT-based viability, confocal, immunoprecipitation (IP), and Western blot (WB) analyses as below.

### 2.2. Cloning of Complementary DNAs

The plasmids for expression of myc-His-tagged wild-type CARP-1 (clone 6.1.2) and CARP-1 anti-sense clone 1.6 were described previously [5]. Additional pcDNA-based plasmids for expression of CARP-1 (Δ600–650), CARP-1 (Δ637–667), EGFP, EGFP-CARP-1 (636–650), and EGFP–H2AX (1–35) were generated by standard molecular biological and cloning manipulations. The open reading frame of the H2AX complementary DNA (cDNA and H2AX cDNA fragments were cloned in pGEX-4T-1 vector to generate Gst-H2AX (full-length), Gst-H2AX (1–35), Gst-H2AX (26–60), Gst-H2AX (47–85), Gst-H2AX (76–110), and Gst-H2AX (101–143) proteins. Additional CARP-1 cDNA fragments were cloned in pTAT-HA vector (22) to generate His-TAT-HA-tagged CARP-1 (552–654), His-TAT-HA-tagged CARP-1 (551–580), His-TAT-HA-tagged CARP-1 (571–600), His-TAT-HA-tagged CARP-1 (591–620), His-TAT-HA-tagged CARP-1 (611–640), His-TAT-HA-tagged CARP-1 (631–660), and His-TAT-HA-tagged CARP-1 (636–650) proteins. All the recombinant plasmids were sequenced to confirm the accuracy and validity of various inserts/epitopes. In addition, His-TAT-HA-tagged CARP-1 (636–652) protein was chemically synthesized to ≥90% purity by Peptides America, Inc. (Fairfax, VA, USA).

### 2.3. Immunoprecipitation (IP), Gst-Pulldown, Western Blot (WB), and MTT Assays

Logarithmically growing cells were either untreated or treated with different agents for various time periods. The cells were lysed to prepare protein extracts. IP was carried out by incubating approximately 1 mg of the protein lysate with appropriate antibodies. The immunoprecipitates were then analyzed by SDS-PAGE, followed by WB with respective, interacting protein. For Gst-pulldown, Gst-H2AX or various His-TAT-HA-tagged CARP-1 peptides were generated in *Escherichia coli* (*E. coli*) BL21 cells essentially as described by us previously [22]. Briefly, the competent *E. coli* BL-21 (DE3) pLysS cells were transformed separately with various recombinant pGEX-4T-1 or pTAT-HA constructs. The transformed cells were then subjected to selection by combined presence of ampicillin and chloramphenicol; various isolates for each transformation were obtained and bacterial lysates were characterized for expression of fusion peptides. Cells from each isolate were grown in 4 mL of LB medium containing ampicillin only until the optical density (OD)_600_ reached 0.4–0.8. Expression of fusion peptides was induced over 2–4 h incubation periods after Isopropyl β-D-1-thiogalactopyranoside (IPTG) addition. The cells were harvested, and pellets were stored at −80 °C overnight. The cell pellets were then lysed in 100–400 μL of bacterial protein extraction reagent (BPER) buffer (Thermo Scientific, Waltham, MA, USA, Cat# 78243) containing 2 μL/mL of 10 units/μL DNAse I (Boehringer Mannheim, Fremont, CA, USA, Cat # 776–785), and supernatant checked for expression of respective fusion peptides by WB. Following confirmation of expression, 5–20 μL of lysate expressing Gst fusion protein was first incubated with 20 μL of precleared glutathione sepharose in a final volume of 100 μL at 4 °C for 2 h with constant rotation. The sepharose beads were spun at 800× *g* for 2 min, and the pellet was washed 2–4 times with 100–200 μL of RIPA buffer (20 mM Tris-HCl (pH 7.5), 150 mM NaCl, 1 mM Na2EDTA, 1 mM EGTA, 1% NP-40, 1% sodium deoxycholate, 2.5 mM sodium pyrophosphate, 1 mM β-glycerophosphate, 1 mM Na_3_VO_4_, 1 µg/mL leupeptin) with 0.5 M NaCl. The beads were spun again as above, and mixed with 5–20 μL of *E. coli* lysate expressing His-TAT-HA CARP-1 peptides. The reactions were incubated further at 4 °C for 2 h with constant rotation. The peptide-bound sepharose beads were pelleted and washed with 100–200 μL of RIPA buffer with 0.1 M NaCl for 2–4 washes. In some cases, additional washes with 0.05 M NaCl buffer were carried out. After the final wash, the sepharose–protein complexes were spun, and then re-suspended in SDS loading buffer for electrophoresis on 12–15% SDS PAGE, followed by WB with appropriate antibodies.

The cell growth inhibition was assessed using an MTT assay following the established protocols described by us previously [5,10,12,13,14,15,16,17,18].

### 2.4. Synthesis and Purification of Peptides

Affinity purification of the Gst-tagged or His-tagged fusion proteins was carried out essentially following procedure previously described by us [22]. Briefly, the bacteria expressing respective fusion protein were cultured in 500 mL of LB medium containing ampicillin until the OD_600_ reached 0.4–0.6. Protein expression was induced by addition of IPTG over an additional 2–4-h incubation at 37 °C. Cells were harvested, and pellets were stored overnight at −80 °C, and lysed in 4–5 mL of BPER lysis buffer containing DNAse I as detailed above. The fusion proteins were then purified utilizing commercially available Gst-sepharose (Pierce Gst purification kit Thermo-fisher Cat#16017) or Ni-NTA columns (HisPur™ Ni-NTA Chromatography Cartridges, ThermoFisher Cat # 90098) following the manufacturer’s suggested methods. Fractions containing eluted fusion protein were pooled, and dialyzed overnight at 4 °C in 1–2 L of 1× Tris-buffered saline (BioRad, Hercules, CA, USA) with multiple buffer changes. Following measurement of protein concentration, the purified protein was aliquoted and snap-frozen in liquid N_2_ for storage. In addition, CARP-1 (636–650) and H2AX (1–35) peptides, each with ≥90% purity, were commercially synthesized followed by labeling of CARP-1 (636–650) with biotin at the amino terminus. We utilized these peptides in the binding studies by surface plasmon resonance as detailed below. Additionally, His-TAT-HA-tagged CARP-1 (636–650) and His-TAT-HA-CARP-1 (636–650 scrambled) peptides with >95% purity were commercially synthesized by Peptides America, Inc., Fairfax, VA, USA.

### 2.5. DNA Damage and Apoptosis Assay

The DNA damage assay was performed using a comet assay kit 96-well plate (Cell Bio Labs, CA, USA) as per the manufacturer’s instructions. Briefly, the cell suspension was mixed with the low-melting agarose at 1:20 ratio and added to the pre-coated 96 well plate. The cells were then subjected to lysis for 1 h, followed by alkaline electrophoresis. The plate was then washed in 70% ethanol and Vista Green DNA dye was added. The comets were visualized in Zeiss LSM780 Confocal microscope. The images were processed using Zeiss ZEN lite software and the comet parameters were measured using Open comet software.

Caspase activities were measured by utilizing the ApoAlert Caspase profiling plate (Clontech, Mountain View, CA, USA) essentially following manufacturer’s suggested guidelines.

### 2.6. Immunofluorescence Staining and Confocal Microscopy

Cells were plated onto chamber slides one day before treatment. Following treatment of cells with respective compounds, the adherent cells were fixed with 5% formaldehyde for 10 min and then washed with phosphate buffered saline (PBS). Samples were blocked (0.5% NP-40, 5% milk powder, 1% fetal bovine serum) for 30 min. After a single wash with PBS, cells were incubated with primary antibodies for 45 to 60 min. Coverslips were washed with PBS and then incubated with secondary antibodies for another 45 to 60 min. They were then washed with PBS and mounted with 0.1 μg/mL 4′,6′-diamidino-2-phenylindole (DAPI) containing mounting solution. For confocal imaging, cells were first fixed with PFA, then stained for CARP-1 by myc-tag antibodies (green), γH2AX antibodies (red), and DAPI (blue) for nuclear staining. Immunofluorescent or confocal images were taken using a Zeiss LSM 510 Meta NLO (63×).

### 2.7. Kinetics of CARP-1–H2AX Interaction

In the first instance, we performed homology modeling of the CARP-1 peptide from positions 631–660 (UniProtKB-Q8IX12; CCAR1_HUMAN) and H2AX peptide from positions 1–35, the region that interacts with CARP-1, (UniProtKB-P16104; H2AX_HUMAN) using SWISS-MODEL [23]. This H2AX region is well characterized due to an existing X-ray structure for histone 2A (PDB ID 1S32) [24]. Protein–protein docking was performed using ZDOCK 3.0.2f with IRaPPA re-ranking [25] to dock the obtained model of CCAR1 with both predicted H2AX models. The top three predictions for each complex, were further subjected to molecular dynamics (MD) using the AMBER14 package to relieve clashes resulting from docking [26]. The ff14SB [27] force field was used for proteins and the TIP3P [28] force field for waters. Each complex was solvated and carefully equilibrated in five stages to ensure proper hydrogen atom orientation. Simulations were conducted for 24 ns at 300 K in a periodic boundary with the NVT ensemble using the Andersen thermostat [29]. The SHAKE algorithm was used to restrain all bonds involving hydrogen. Each trajectory was analyzed using CPPTRAJ [30] and was subjected to MM-GBSA/PBSA [31] analysis to evaluate the strength of the protein–protein interaction.

Next, kinetics of CARP-1 binding with H2AX were determined by surface plasmon resonance (SPR) technology (Profacgen, Shirley, NY, USA). Briefly, CARP-1 (636–650) peptide was dissolved in water, and various concentrations of CARP-1 peptide were manually printed onto the bare gold-coated (thickness 47 nm) PlexArray Nanocapture Sensor Chip at 40% humidity. The sensor chip was obtained from Plexera Bioscience (Seattle, WA, USA). Each concentration was printed in replicate, and each spot contained 0.2 μL of sample solution. The chip was incubated in 80% humidity at 4 °C for overnight, and rinsed with 10× PBST for 10 min, 1× PBST for 10 min, and deionized water twice for 10 min. The chip was then blocked with 5% (*w/v*) non-fat milk in water overnight, and washed with 10× PBST for 10 min, 1× PBST for 10 min, and deionized water twice for 10 min, before being dried under a stream of nitrogen prior to use. The binding reactions were performed in PBST buffer (0.01 M phosphate-buffered saline (0.138 M NaCl; 0.0027 M KCl), 0.05% Tween-20, pH 7.4. SPRi measurements were performed with PlexAray HT (Plexera Bioscience, Seattle, WA, USA). Collimated light (660 nm) passes through the coupling prism, reflects off the SPR-active gold surface, and is received by the CCD camera. Buffers and samples were injected by a non-pulsatile piston pump into the 30-μL flow cell that was mounted on the coupling prim. Each measurement cycle contained four steps: washing with PBST running buffer at a constant rate of 2 μL/s to obtain a stable baseline, sample injection at 5 μL/s for binding, surface washing with PBST at 2 μL/s for 300 s, and regeneration with 0.5% (*v/v*) H_3_PO_4_ at 2 μL/s for 300 s. All the measurements were performed at 25 °C. The signal changes after binding and washing (in AU) were recorded as the assay value. Selected protein-grafted regions in the SPR images were analyzed, and the average reflectivity variations of the chosen areas were plotted as a function of time. Real-time binding signals were recorded and analyzed using a data analysis module (DAM) obtained from Plexera Bioscience (Seattle, WA, USA). Kinetic analysis was performed using BIAevaluation 4.1 software from Biacore, Inc. (New York, NY, USA).

Association and dissociation rate constants were calculated by numerical integration and global fitting to a 1:1 interaction model and the equation, dRU*(t)/dt* = k_a_C(R_max_ − RU(*t*)) − k_d_RU(*t*), where RU(*t*) is the response at time *t*, R_max_ is the maximum response, C is the concentration of analyte in solution, k_a_ is the association rate constant, k_d_ is the dissociation rate constant, and RU (0) = 0.

### 2.8. Statistical Analyses

The statistical analyses were performed using Prism 6.0 software. The data were expressed as means ± SEM and analyzed using a two-tailed Student’s *t*-test or one-way ANOVA followed by a post hoc test. A *p*-value < 0.05 was considered statistically significant.

## 3. Results

### 3.1. Adriamycin or CFMs Inhibit Cell Growth, in Part by Stimulating CARP-1 Expression, Phosphorylation of JNKs and H2AX, and DNA Damage

**Figure 1:**
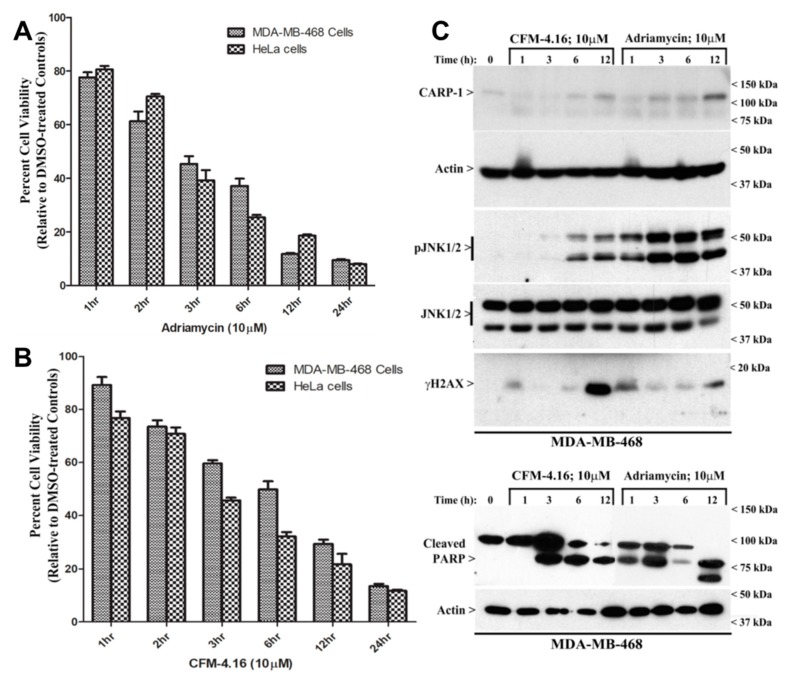
Adriamycin and CFM-4.16 inhibit cell growth, elevate CARP-1 expression, and induce activation of JNKs and H2AX, and apoptosis. Noted cell lines were either treated with DMSO (Control), Adriamycin (**A**), or CFM-4.16 (**B**) for indicated dose and time. Cell viability was determined by MTT assay. The columns in the bar charts represent means of three independent experiments; bars, SE. (**C**) MDA-MB-468 cells were either untreated (noted as 0), or treated with Adriamycin or CFM-4.16 for noted dose and time. Cell lysates were analyzed with WB as described in Section 2 for levels of CARP-1, phospho- and total JNK1/2, γH2AX, and cleaved PARP. The WB membranes were subsequently probed with anti-actin antibodies to assess equal loading. In panel C, representative autorads out of two independent experiments is presented. The presence of respective protein is indicated by an arrowhead on the left side of each blot. The approximate location of various molecular-weight markers is indicated on the right side of each blot; kDa, kilodalton.

A number of our prior studies revealed growth inhibition of a variety of cancer cells by Adriamycin or the CFM class of compounds [5,10,12,13,14,15,16,17,18]. Here, we utilized human TNBC MDA-MB-468 and human cervical cancer HeLa cells to conduct an MTT-based dose-response analysis using Adriamycin and CFM compounds. We chose these and other cancer cell lines (see Figure 3 below) to demonstrate that the DNA damage-induced apoptosis signaling mechanism(s) are functional in cells of different histological/cancer type and not selective for a particular cell line. Since Adriamycin, CFM-4, and CFM-4.16 have molecular masses of 543.3, 404.9, and 440.3, respectively, we chose to use an equimolar dose of each compound in the MTT assays. A dose-response analysis indicated a robust cell-growth inhibition by a 10 μM dose of Adriamycin in MDA-MB-468 cells (Figure S1A). Since our prior studies showed that a 10 μM dose of CFM-4.16 also induced a robust inhibition of growth of MDA-MB-468 cells [16], we utilized a 10 μM dose of each compound for growth inhibition and WB experiments. A 10 μM dose of either of these compounds inhibited viabilities of both the cell lines in a time-dependent manner (Figure 1A,B and Figure S1A, Supplementary Materials). Prior reports established that phosphorylation of H2AX at serine 139 (γ-H2AX) is involved in DNA damage repair and apoptotic signaling by ATM/ATR and JNKs, respectively [19,20,21]. Next, the MDA-MB-468, HeLa, and the kidney cancer UOK 262 cells were treated with a 10 μM dose of the CFM-4.16 or Adriamycin for various time periods. The cell lysates from the control, untreated, and treated cells were analyzed by WB for expression of CARP-1, phospho-JNK1/2, phospho-H2AX, and poly ADP ribose polymerase (PARP) proteins. As shown in Figure 1C and Figure S1B,C (Supplementary Materials), both the CFM-4.16 and Adriamycin stimulated expression of CARP-1, phospho-JNK1/2, and phospho-H2AX in a time-dependent manner in all the three cell lines. Please note that Adriamycin-induced H2AX activation was robust at 12 h in the treatment period. This H2AX activation by Adriamycin is expected and correlates with current understanding that suggests activation of H2AX by ATM/ATR kinases often signals DNA repair during low to moderate levels of DNA damage that will contribute to the cell’s ability to overcome damage and survive [19]. The H2AX activation is also indicative of DNA damage and involves pro-apoptotic JNKs [20,21]. Our data in Figure 1C and Figure S1B,C (Supplementary Materials) also show activation of JNKs; however, a robust JNK activation is co-incident with H2AX phosphorylation following treatments with Adriamycin or CFM-4.16. Treatment of MDA-MB-468 cells with either of the compounds also provoked PARP cleavage in a time-dependent manner, with a robust cleaved PARP noted over a 12-h treatment period, although Adriamycin stimulated PARP cleavage as early as 3 h of treatment (Figure 1C). These findings collectively demonstrate that both the compounds inhibit cell growth in part by stimulating apoptosis, and a robust apoptosis activation occurs at 12 h of treatments with respective compounds, co-incident with a robust increase in CARP-1 levels and activities of JNKs and H2AX.

**Figure 2:**
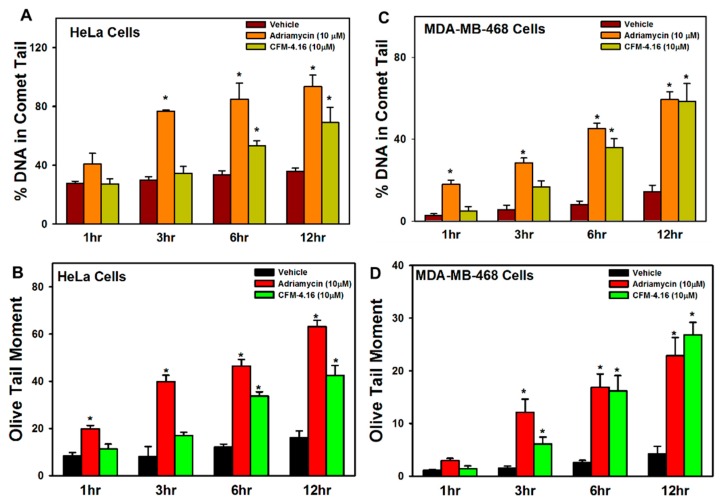
Adriamycin and CFM-4.16 induce DNA damage. Cells were treated with the noted dose of respective compound for indicated time periods. Mean values for percentage tail DNA content (**A**,**C**) and Olive tail moment (**B**,**D**) for respective cells from control and treatment periods for each compound were measured using software recommended by the manufacturer of the kit for measurement of DNA damage (Cell Bio Labs, CA, USA). The columns in each chart indicate average values for respective treatment condition; bars, SE; * *p* ≤ 0.05.

Adriamycin is well known to induce DNA damage. Given the critical role of CARP-1 in apoptotic signaling by Adriamycin and CFM compounds, and the fact that stress-activated protein kinases (SAPKs) p38 and JNKs are activated in apoptosis signaling by CFM-4.16 [6,12,13,14,15,16,17,18], we next determined whether CFM-4.16 also provoked DNA damage. We treated MDA-MB-468 and HeLa cells with a 10 μM dose of Adriamycin or CFM-4.16 for 1, 3, 6, and 12 h, followed by assessment of damaged DNA by the comet assay as described in Section 2. The presence of comet-like tails that contain the fragmented cellular DNA is a well-known marker/indicator of apoptosis [32]. We measured the comet parameters such as percentage tail DNA and mean tail moment. As shown in the Figure 2A–D, Table S1, and Figure S2A,B (Supplementary Materials), the percentage of tail DNA, as well as tail moment, significantly increased in cells treated with Adriamycin or CFM-4.16 in a time-dependent manner. Of note is the fact that Adriamycin and related anthracyclines intercalate between DNA bases and trap topoisomerase II (TopoII) in the double-strand cleavage form, thus introducing double-strand breaks (DSBs) due to TopoII poisoning [33]. It remains to be clarified whether CFM-4.16 also targets cellular DNA and/or the associated DNA repair complex protein(s) to introduce DNA strand breaks, or activates caspase-activated DNAses to breakdown cellular genomic DNA. Nevertheless, our data in Figure 1 and Figure 2 suggest that, similar to promotion of DNA damage by Adriamycin and CFM-4.16, molecular mechanisms of H2AX-dependent apoptosis signaling likely overlap between the two agents.

### 3.2. CARP-1 Interacts with H2AX, and Knock-Down of CARP-1 Leads to Reduced H2AX in Cells Treated with Adriamycin or CFM-4.16

**Figure 3:**
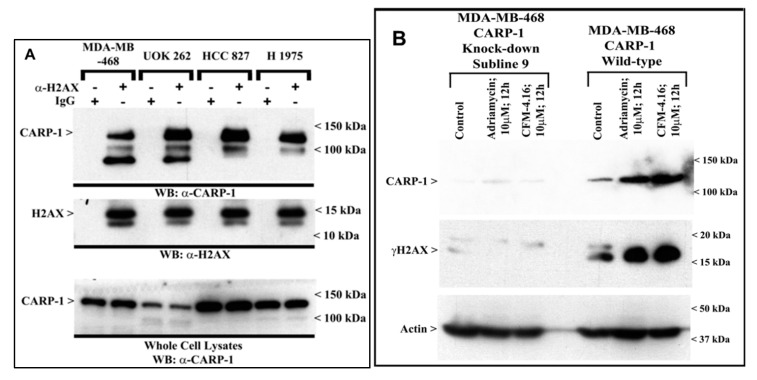
CARP-1 interacts with H2AX, and knock-down of CARP-1 abrogates γH2AX following treatments with Adriamycin or CFM-4.16. (**A**) Approximately 1 mg of cell lysate from each of the indicated cells was subjected to immunoprecipitation using anti-H2AX antibodies. The immunoprecipitates were then analyzed with WB by probing the membrane with anti-CARP-1 (α2) antibodies (top autoradiograph). The membrane was subsequently re-probed with anti-H2AX antibodies to ascertain presence of H2AX (middle autoradiograph). The whole-cell lysates were separately analyzed with WB to determine endogenous CARP-1 in the respective cell line (Lower autoradiograph). (**B**) Cells were either untreated (Control), or treated with Adriamycin or CFM-4.16 for noted dose and time. Cell lysates were analyzed with WB as described in Section 2 for levels of CARP-1 and γH2AX. The WB membrane was subsequently probed with anti-actin antibodies to assess equal loading. The presence of respective proteins is indicated by an arrowhead on the left side of each blot. The approximate location of various molecular-weight markers is indicated on the right side of each blot; kDa, kilodalton.

Since CARP-1 depletion prevented apoptosis due to Adriamycin or the CFM compounds [5,7,10,12,13,14,15,16,17,18], we tested whether CARP-1 interacted with H2AX and the extent to which this interaction regulated H2AX activation during apoptosis signaling. Co-immunoprecipitation and WB studies with lysates from MDA-MB-468, UOK262, and non-small-cell lung cancer (HCC827 and H1975) cells revealed CARP-1 interaction with H2AX (Figure 3A). We next clarified whether CARP-1 is required for H2AX activation. As shown in WB analyses in Figure 3B, Figure S3A, and immunofluorescence (IF) analysis in Figure S3B (Supplementary Materials), while Adriamycin or CFM-4.16 caused a robust increase in CARP-1 levels and γH2AX in MDA-MB-468 and HCC827cells, knock-down of CARP-1 abrogated CARP-1 and γH2AX increase by either of the compounds, suggesting a requirement for CARP-1 in activation of H2AX.

### 3.3. CARP-1 (636–650) Binds with H2AX (1–35)

**Figure 4:**
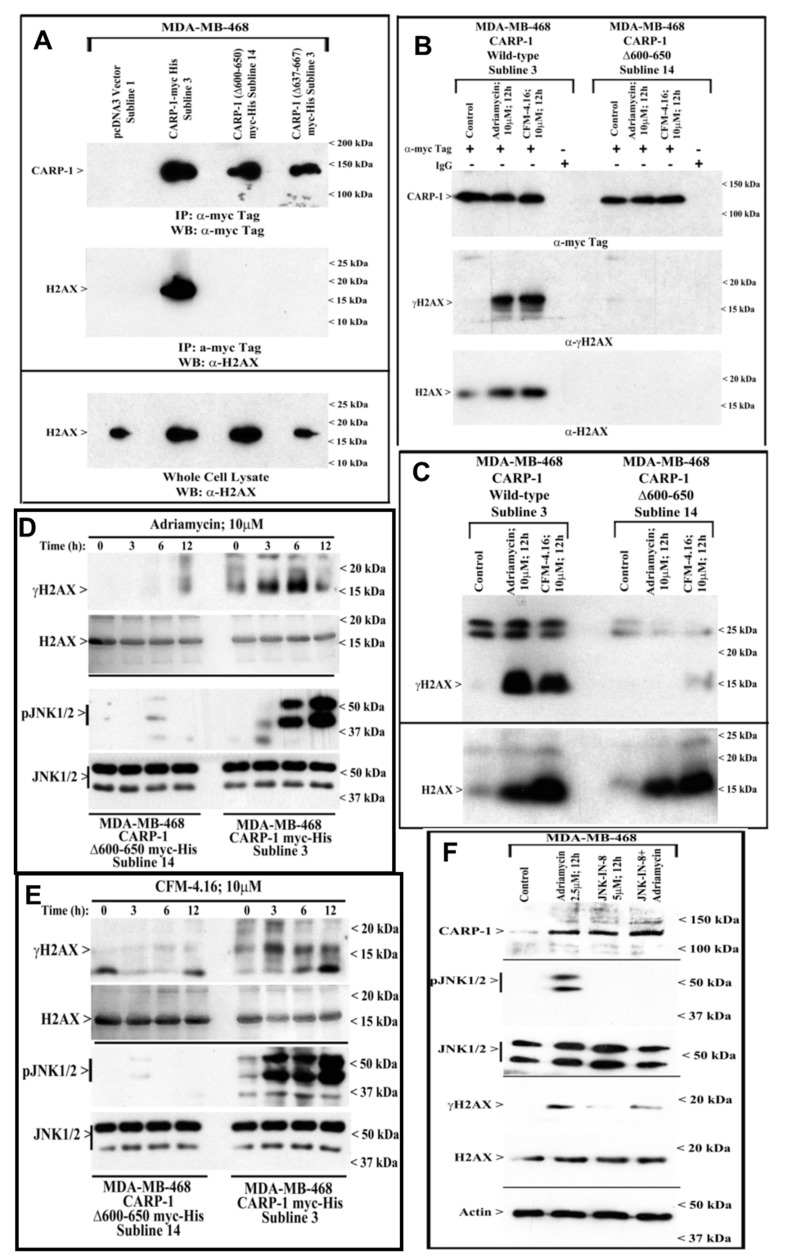
Deletion of CARP-1 amino acids 600–650 abrogates H2AX–CARP-1 interaction, and activation of H2AX and JNKs by Adriamycin or CFM-4.16. (**A**) Approximately 1 mg of cell lysate from each of the indicated cells was subjected to immunoprecipitation using anti-myc tag antibodies. The immunoprecipitates were then analyzed with WB by probing the membrane with anti-myc tag (for CARP-1) (top autoradiograph). The membrane was subsequently re-probed with anti-H2AX antibodies to ascertain presence of H2AX (middle autoradiograph). The whole-cell lysates were separately analyzed with WB to determine endogenous H2AX in the respective cell line (Lower autoradiograph). (**B**) Cells stably expressing myc-His-tagged CARP-1 or its Δ600–650 mutant were either untreated (Control), or treated with Adriamycin or CFM-4.16 for noted dose and time. Cell lysates were analyzed by immunoprecipitation using anti-myc tag or immunoglobulin G (IgG), followed by WB analysis using anti-myc tag (for CARP-1), γH2AX, or H2AX antibodies. (**C**) Cells were treated as in (**B**), and lysates were analyzed with WB using γH2AX and H2AX antibodies. (**D**,**E**) Cells stably expressing myc-His-tagged CARP-1 or its Δ600–650 mutant were either untreated (0), or treated with Adriamycin or CFM-4.16 for noted dose and time. Cell lysates were analyzed with WB using anti-γH2AX or phospho-JNK1/2 antibodies followed by probing of the respective membranes with antibodies for total H2AX and JNK1/2. myc tag (for CARP-1), or γH2AX antibodies. (**F**) MDA-MB-468 cells were either untreated (Control), or treated with noted agents. Cell lysates were analyzed with WB as described in Section 2 for levels of CARP-1, phospho- and total JNK1/2, γH2AX, and H2AX. The WB membranes were subsequently probed with anti-actin antibodies to assess equal loading. The presence of respective proteins in (**A**–**F**) is indicated by an arrowhead on the left side of each blot. The approximate location of various molecular-weight markers is indicated on the right side of each blot; kDa, kilodalton.

We next carried out mutagenesis-based studies to map the interacting epitopes of CARP-1 and H2AX proteins. In the first instance, we generated multiple, neomycin-resistant, MDA-MB-468 stable cell lines expressing pcDNA3 vector, myc-His-tagged wild-type CARP-1, or CARP-1 mutants harboring in-frame deletion of CARP-1 amino acids 600–650 or 637–667 essentially as detailed in Section 2 and our prior publication [6]. The CARP-1 interaction with H2AX was then investigated by immunoprecipitation and WB analyses. As shown in Figure 4A, H2AX interacted with wild-type CARP-1 but not either of the CARP-1 mutants that harbored deletion of amino acids 600–650 or 637–667, suggesting that the H2AX-interacting epitope of CARP-1 likely resided within amino acids 637–650. Additional immunoprecipitation and WB analyses revealed that, while Adriamycin or CFM-4.16 treatments induced a robust interaction of wild-type CARP-1 with γH2AX, in-frame deletion of CARP-1 amino acids 600–650 abrogated CARP-1 interaction with γH2AX (Figure 4B,C). Furthermore, consistent with our findings in Figure 1, treatments with Adriamycin or CFM-4.16 caused a time-dependent stimulation of JNK1/2 and H2AX activation in cells overexpressing myc-His-tagged CARP-1 (Figure 4D,E). However, treatments with either of the compounds failed to activate JNK1/2 or H2AX in cells stably expressing CARP-1 mutant with in-frame deletion of amino acids 600–650 (Figure 4D,E, Figure S4, Supplementary Materials). Although inhibition of CARP-1 binding with H2AX abrogated activation of JNK1/2 by Adriamycin or CFM-4.16 (Figure 4D,E), we next clarified whether activation of JNK1/2 was required for H2AX phosphorylation. For this purpose, we utilized JNK-IN-8, an allosteric inhibitor of JNK activation, and utilized a 5 μM dose of this compound for cell viability and protein expression studies essentially as described previously [34]. Treatments of MDA-MB-468 cells with Adriamycin, JNK-IN-8, or a combination caused elevated levels of CARP-1, while exposure to JNK-IN-8 resulted in total loss of JNK activation (Figure 4F). Adriamycin treatment, however, caused a robust increase in γH2AX, while combined presence of JNK-IN-8 and Adriamycin resulted in reduced levels of γH2AX (Figure 4F). Interestingly, exposure to JNK-IN-8 alone also stimulated an increase in CARP-1 levels. Although MDA-MB-231 HBC cells that were treated with JNK-IN-8 had significantly reduced viabilities when compared with their untreated counterparts [34], whether elevated CARP-1 in cells treated with JNK-IN-8 occurs due to inhibition of JNKs or potential, off-target stress-inducting effects remains to be clarified. Nevertheless, these data collectively demonstrate that CARP-1 interaction with H2AX is likely necessary for activation of JNK1/2 and H2AX by Adriamycin or CFM-4.16, and JNK activation is required for Adriamycin-dependent phosphorylation/activation of H2AX. Moreover, Adriamycin induces expression of CARP-1 and a faint activation of JNK1/2 and H2AX seems to occur in CARP-1 (Δ600–650) expressing cells following treatments with Adriamycin or CFM-4.16 (Figure 4C,D), and this is likely due to the endogenous CARP-1-JNK1/2-H2AX signaling. However, since overall JNK1/2 and H2AX activation by Adriamycin or CFM-4.16 is diminished in cells overexpressing CARP-1 (Δ600–650) when compared with their wild-type expressing counterparts (Figure 4C,D), it is possible that Adriamycin induction of endogenous CARP-1–JNK1/2–H2AX apoptosis signaling is insufficient in promoting apoptosis in CARP-1 (Δ600–650) cells. Collectively, overexpression of CARP-1 (Δ600–650) would block Adriamycin-dependent JNK1/2 activation and, in turn, likely functions to promote survival.

**Figure 5:**
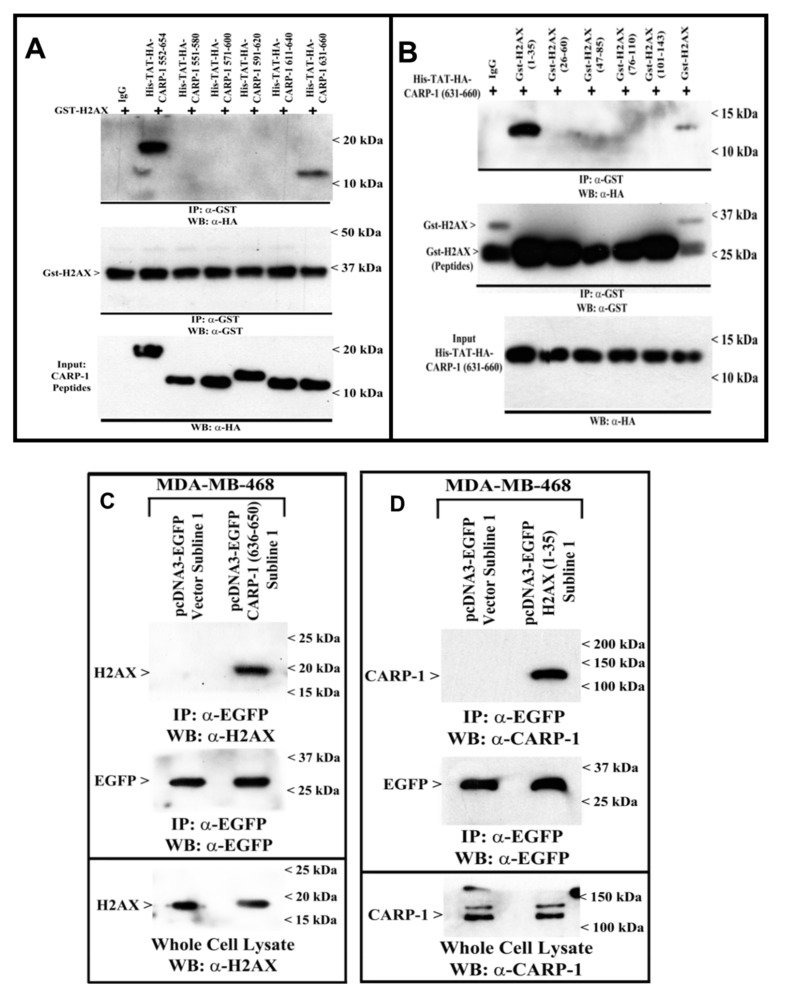
Fine Mapping of H2AX/CARP-1 interacting epitopes. The Gst-tagged H2AX protein, and various Gst-tagged H2AX peptides and His-TAT-HA-tagged CARP-1 peptides were purified following expression in *E. coli* BL-21 cells essentially as described in Section 2. (**A**) Gst-H2AX protein was immobilized on glutathione sepharose followed by incubation indicated CARP-1 peptides. Following stringent washing, the bound proteins were analyzed with WB using anti-HA (upper) or anti-Gst (middle) antibodies. The lower blot shows respective HA-tagged CARP-1 peptides used as input. (**B**) Indicated Gst-tagged H2AX peptides were firstly immobilized on glutathione sepharose followed by incubation with His-TAT-HA-CARP-1 (631–660) peptide. Stringent washing and WB analyses were performed as in panel A. (**C,D**) Approximately 1 mg of cell lysate from each of the indicated cells stably expressing EGFP, EGFP-CARP-1 (636–650), or EGFP-H2AX (1–35) was subjected to immunoprecipitation using anti-EGFP antibodies. The immunoprecipitates were then analyzed with WB by probing the membrane with anti-EGFP, anti-H2AX, or anti-CARP-1 (α2) antibodies as noted in the upper boxes in (**C**,**D**). The whole-cell lysates were separately analyzed with WB to determine endogenous H2AX and CARP-1 levels in the respective cell line (lower boxes in (**C**,**D**)). The presence of respective proteins is indicated by an arrowhead on the left side of each blot. The approximate location of various molecular-weight markers is indicated on the right side of each blot; kDa, kilodalton.

We further clarified whether CARP-1 bound to H2AX directly by utilizing various *E. coli*-expressed His-TAT-HA-tagged CARP-1 and Gst-tagged H2AX peptides as detailed in Section 2. The data revealed that His-TAT-HA-CARP-1 (552–654) and His-TAT-HA-CARP-1 (631–660) bound with Gst-H2AX protein (Figure 5A), while His-TAT-HA-CARP-1 (631–660) peptide bound with Gst-H2AX and Gst-H2AX (1–35) proteins (Figure 5B). Together with data in Figure 4B, our mutagenesis and peptide-binding studies demonstrate that CARP-1 (636–650) and H2AX (1–35) regions harbor minimal epitopes necessary for CARP-1–H2AX binding. We then generated MDA-MB-468 and Hela sublines that stably express EGFP-tagged H2AX (1–35) or EGFP-tagged CARP-1 (636–659) fusion proteins (Figure S5A,B, Supplementary Materials) to demonstrate in-cell interaction of the CARP-1 (636–650) peptide with endogenous H2AX, and H2AX (1–35) peptide with endogenous CARP-1. Cell lysates from the MDA-MB-468 sublines that stably express EGFP vector or EFGP-CARP-1 (636–650) were first subjected to immunoprecipitation using anti-EGFP antibodies followed by WB analysis using anti-H2AX antibodies. Figure 5C shows a robust, in vivo (in-cell) interaction of CARP-1 (636–650) with endogenous H2AX protein. In an analogous experiment, MDA-MB-468 sublines that stably express EGFP vector or EFGP-H2AX (1–35) were subjected to immunoprecipitation using anti-EGFP antibodies followed by WB analysis using anti-CARP-1 (α2) antibodies. Figure 5D shows a robust, in vivo interaction of H2AX (1–35) with endogenous CARP-1.

### 3.4. Kinetics of CARP-1 (636–650) Binding with H2AX (1–35)

**Figure 6:**
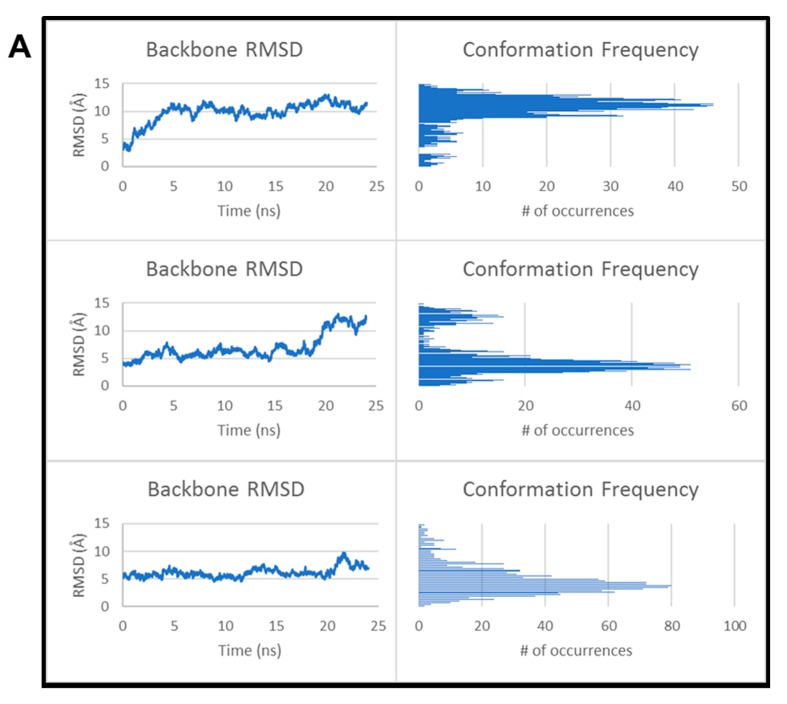

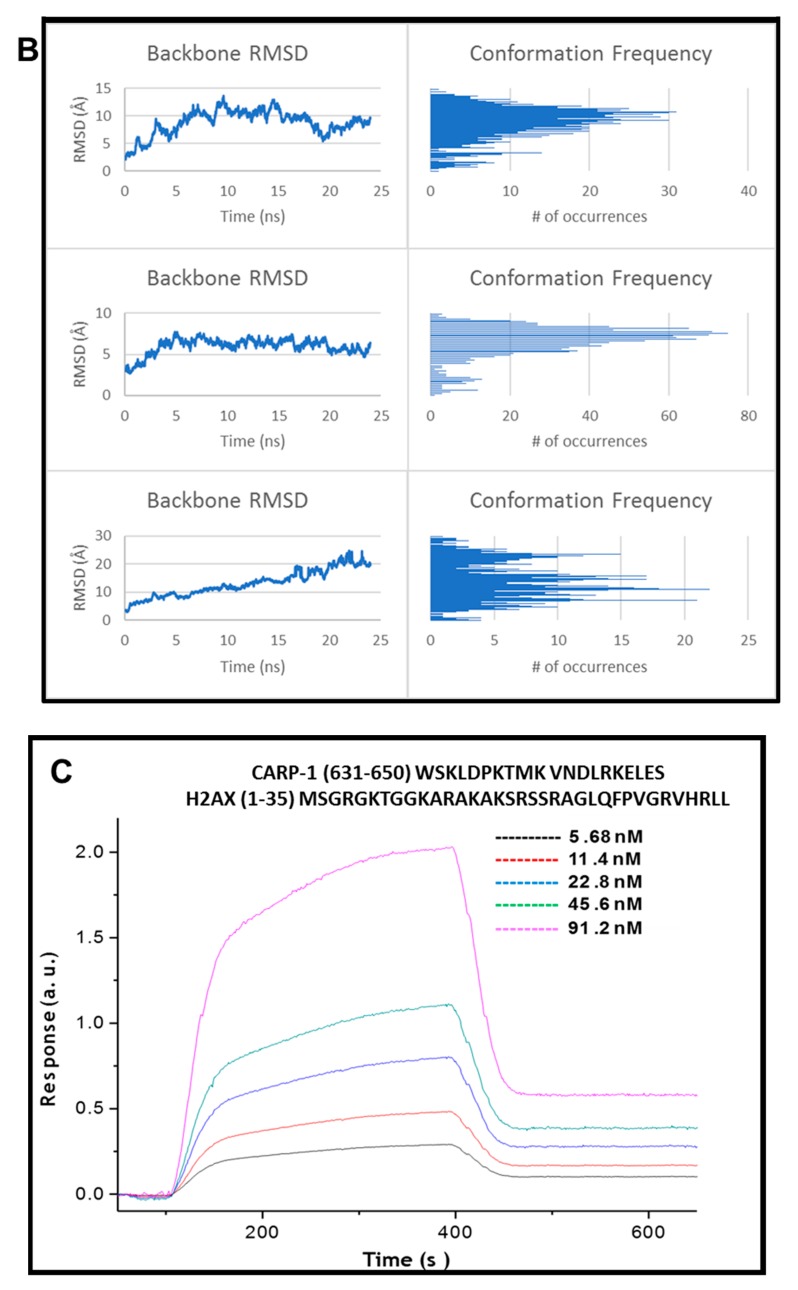
Computational analyses of H2AX (1–35) binding with CARP-1 (631–650). Backbone RMSD calculations and conformation frequency analysis for the three poses (top, middle, and lower boxes) of the docked CARP-1 (model A)/H2AX complex (**A**), and CARP-1 (model B)/H2AX complex (**B**). Each analysis was completed over 24 ns of simulation for each complex. (**C**) Surface plasmon resonance sensogram showing binding of noted CARP-1 and H2AX peptides (inset) as detailed in Section 2.

We conducted computational modeling and surface plasmon resonance studies to investigate the binding kinetics of CARP-1 and H2AX peptides. We firstly performed computational modeling studies utilizing various models and algorithms in conjunction with CARP-1 residues 631–660 and H2AX residues 1–35 as detailed in Section 2. Although a crystal structure of CARP-1 is yet to be resolved, the structural prediction using SWISS-MODEL (Figure S6A, Supplementary Materials) indicated 48% homology to the SAP domain of E1B-55kDa-associated protein 5 (PDB ID: 1ZRJ; [35]) The crystal structure of H2AX, on the other hand, is well characterized. Based on an existing H2AX X-ray structure, two structural models were produced (Figure S6B, Supplementary Materials). The computational analyses were then conducted to perform protein–protein docking using the model of CARP-1 with both predicted H2AX models as detailed in Section 2. The conformational stability of each complex was then analyzed as, presumably, the more tightly bound or constrained a complex, the less fluxionality might be expected. Furthermore, relieving steric clashes should increase the backbone root mean square deviation (RMSD) significantly when considering this small structure. This was reflected in the backbone RMSD throughout the simulation (Figure 6 and Figure S6, Supplementary Materials). Even after equilibration and heating, the backbone RMSD was significantly higher than it would be for an entire protein that would otherwise have less backbone flux [36,37]. Complex 1 of CARP-1/H2AX model A (Figure 6A; top row) initially showed a very small change in structure; however, it quickly adopted a very different conformation from the initial structure. This was reflected by the large, steady RMSD. In complex 2 (Figure 6A; middle row), two different conformations occurred. Initially, the protein was stable with a backbone RMSD of 5.5 Å compared to the starting structure. After 20 ns, it began to adopt a significantly different conformation as can be seen by the presence of a second spike in the conformation frequency. The third complex (Figure 6A; bottom row) was stable throughout the entire simulation as indicated by the presence of only one dominant conformer. Most importantly, all three complexes remained bound throughout the entire simulation. We also analyzed the CARP-1 complex with H2AX model B (Figure 6B). Except for the third pose (bottom row) in Figure 6B, a similar trend was observed for top and middle rows as noted for the CARP-1/H2AX model in Figure 6A complexes. The top pose in Figure 6B displays more conformational instability as seen through the larger number of peaks observed in the histogram, although there is one dominant conformation observed at an RMSD of 10.4 Å. The middle complex in Figure 6B was much more stable throughout the simulation with a single dominant conformer at approximately 5.9 Å RMSD, as also was the third complex in the bottom row of Figure 6B. By contrast, the third pose was very unstable as shown through not only the large number of conformations, but also through the steadily increasing RMSD. In this simulation, the two peptides dissociated from each other [35] resulting in the increasing RMSD. We did not consider this pose in subsequent MM-PBSA/GBSA analysis.

To determine whether these two proteins have potential to interact in a biological setting, we subjected each complex to MM-PBSA/GBSA analysis (Table 1). Calculations were computed from 200 poses within the last 5 ns of the simulation. Due to the larger flexibility afforded to small peptides compared to an entire protein, the energy calculated at each pose can be significantly different compared to nearby poses. This was reflected in the large standard deviations observed for each complex. However, the binding energies were relatively close to each other and of significantly high magnitude to suggest that protein–protein interaction between these two regions of CARP-1 and H2AX is possible. Absolute binding energies were shown across the different complexes; however, these values were not exact and only served as a comparison to each other for reference.

The predicted kinetics of interaction of CARP-1 (631–660) and H2AX (1–35) epitopes was further validated by utilizing respective, chemically synthesized peptides to determine their in-solution binding by surface plasmon resonance technology as described in Section 2. As shown in Figure 6C, this experiment revealed an equilibrium dissociation constant (K_d_ Value) of 1.26 × 10^−7^ M. (K_a_ = 2.32 × 10^3^ M^−1^·s^−1^, K_d_ = 2.92 × 10^−4^·s^−1^). Collectively, our data in Figure 5 and Figure 6 demonstrate that binding of CARP-1 and H2AX proteins involved a single, respective epitope, and that binding of the CARP-1 (636–650) and H2AX (1–35) regions was direct and strong.

### 3.5. Interference with CARP-1–H2AX Binding Abrogates Cell-Growth Inhibition by Adriamycin or CFM Compounds

**Figure 7:**
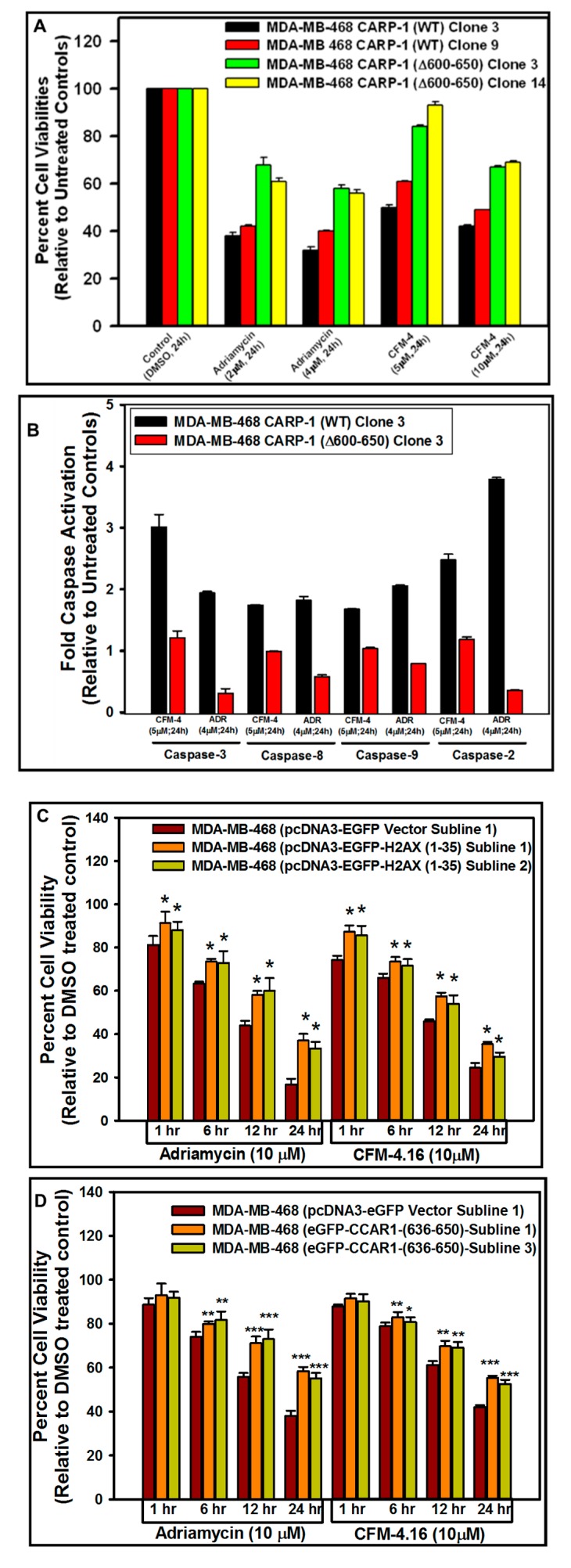

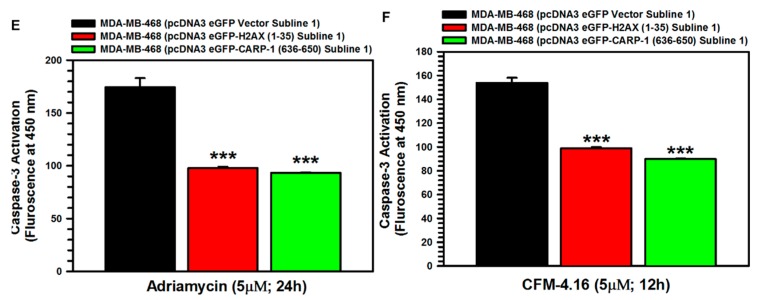
Disruption of H2AX interaction with CARP-1 results in enhanced viabilities of CFM-4-, CFM-4.16-, or Adriamycin-treated cells in part due to reduced apoptosis. (**A**,**C**,**D**) Indicated MDA-MB-468 cell lines were treated with DMSO (Control), or with the noted dose and time of Adriamycin, CFM-4, or CFM-4.16 compounds. Determination of viable/live cells was carried out by MTT assay as described in Section 2. (**B**,**E**,**F**) Cell lysates derived from vehicle DMSO (Control), or CFM-4-, CFM-4.16-, or Adriamycin-treated cells were added to the wells that had immobilized fluorogenic substrates of noted caspases. The fluorescence released from the activated caspase-dependent cleavage of respective substrate was detected by a plate reader at the excitation and emission wavelengths of 380 nm and 460 nm, respectively, as detailed in Section 2. The columns in bar charts in panels A, C, D and B, D, F represent means of three and two independent experiments, respectively; bars, SE. For panels C and D, * *p* < 0.05, **p < 0.01 and *** *p* ≤ 0.001 relative to the respective EGFP vector subline. For panels E and F, *** *p* ≤ 0.05 relative to the respective EGFP vector subline.

Our next objective was to analyze the biological significance of the CARP-1–H2AX interaction in cells. For this purpose, we utilized MDA-MB-468 and HeLa cell lines that stably express myc-His-tagged wild-type CARP-1 or CARP-1 (Δ600–650)-myc-His proteins as detailed in Figure S4 (Supplementary Materials). These sublines were then either untreated (Control, DMSO), or treated with Adriamycin or CFM-4 compound. Cells expressing CARP-1 (Δ600–650) mutant were resistant to inhibition by CFM-4 or ADR when compared with their WT CARP-1-expressing counterparts (Figure 7A and Figure S7A, Supplementary Materials). Next, we measured activation of caspases as an apoptosis readout in cells expressing myc-His-tagged wild-type CARP-1 or CARP-1 (Δ600–650) proteins that were either untreated or treated with Adriamycin or CFM compounds as detailed in Section 2. We utilized a lower, 4–5 μM dose of Adriamycin or CFM compounds because a 10 μM Adriamycin dose caused interference in signal output/readout in this particular assay, likely due to background fluorescence that is emitted by higher dose of Adriamycin. Although Adriamycin has a maximum excitation and emission wavelength of 470 and 560 nm, respectively [38], we found significant fluorescence emission in cells treated with 10 μM Adriamycin at a 380-nm excitation wavelength. Moreover, since caspase activation precedes apoptotic cell death, we utilized a lower dose of Adriamycin to permit a robust measurement of optimal caspase activation in cells undergoing moderate apoptosis with lower/minimal interference by Adriamycin-induced fluorescence signals. Our data revealed that there was an overall reduction in activation of caspases-3, -8, -9, and -2 in Adriamycin-treated CARP-1 (Δ600–650) cells when compared with their wild-type CARP-1-expressing counterparts (Figure 7B). We then clarified whether stable expression of CARP-1 (636–650) or H2AX (1–35) peptides, which in principle will compete for the endogenous CARP-1-H2AX interaction, would also abrogate inhibitory effects of Adriamycin or CFM compounds. Here, we utilized MDA-MB-468 and HeLa cell lines that stably express EGFP, EGFP-CARP-1 (636–650), or EGFP-H2AX (1–35) proteins, as described in Figure 5 above. Cells expressing EGFP-CARP-1 (600–650) or EGFP-H2AX (1–35) fusion proteins were resistant to inhibition by CFM-4.16 or ADR when compared with their respective, EGFP-expressing counterparts (Figure 7C,D and Figure S7B,C, Supplementary Materials) that again was due in part to an overall reduction in activation of caspase-3 (Figure 7E,F). Further flow-cytometric analysis of the MDA-MB-468 cells expressing EGFP or EGFP-CARP-1 (636–650) that were either untreated (control), or treated with Adriamycin or CFM-4.16 were conducted to monitor apoptosis. As shown in Figure S7D (Supplementary Materials), Adriamycin or CFM-4.16 treatments resulted in a generally higher percentage of EGFP-expressing cells undergoing apoptosis when compared with similarly treated EGFP-CARP-1 (636–650)-expressing counterparts. Whether competition for the endogenous CARP-1–H2AX interaction by stably expressed H2AX (1–35) peptide altered H2AX activation and/or translocation to confer resistance to Adriamycin was next clarified by confocal analyses. MDA-MB-468 or HeLa cells that express EGFP or EGFP-H2AX (1–35) were separately treated with Adriamycin, and presence of CARP-1 and γH2AX was determined by immuno-cytochemical staining followed by photography by a confocal microscope as described in Section 2. Hyper-phosphorylation of H2AX by JNKs and consequent γH2AX cytoplasmic distribution was previously shown to promote apoptosis following DNA damage [19,20,21]. The confocal analyses shown in Figure S6E (Supplementary Materials) revealed increased nuclear presence of γH2AX in Adriamycin-treated, cells that express EGFP-H2AX (1–35) when compared with their EGFP-expressing, Adriamycin-treated counterparts. Since nuclear presence of γH2AX is often associated with DNA damage repair, it is likely then that the nuclear presence of γH2AX in EGFP-H2AX (1–35) cells contributes to more effective repair of Adriamycin-induced DNA damage and, in turn, promotes cell survival in the presence of Adriamycin when compared with the EGFP-expressing, Adriamycin-treated cells.

**Figure 8:**
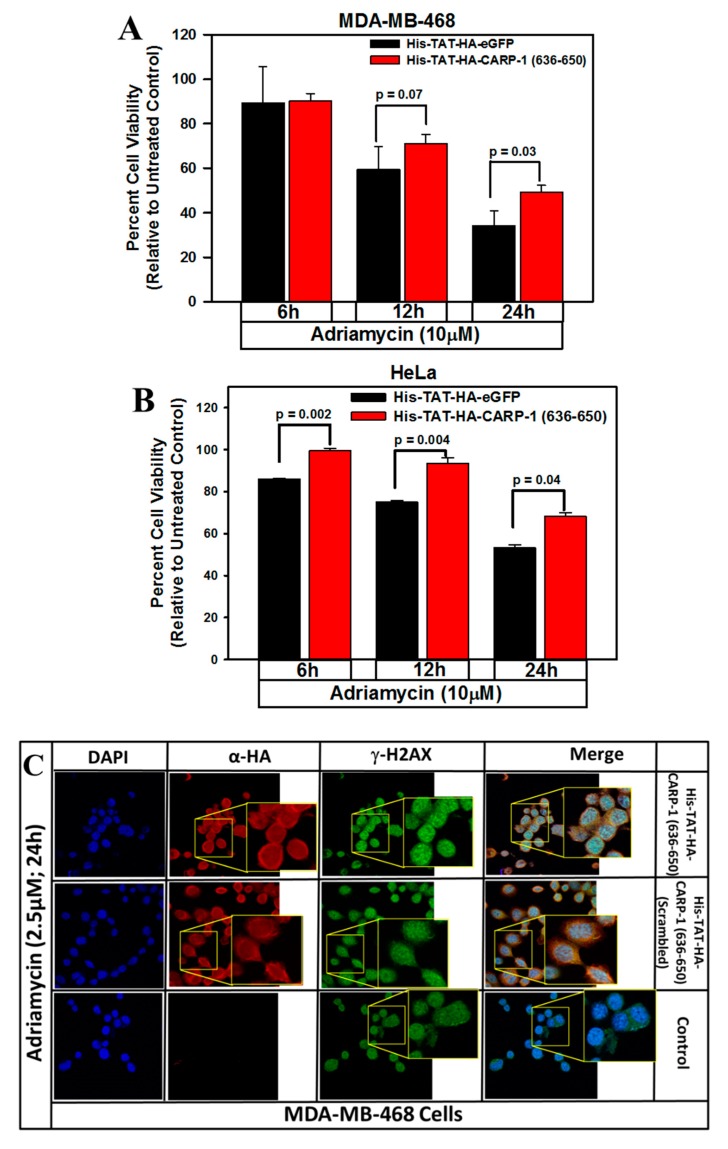
Ectopic expression of TAT-CARP-1 (636–650) peptide results in enhanced viabilities of Adriamycin-treated cells. MDA-MB-468 (**A**) or HeLa (**B)** cells were pre-incubated with 300 μg/mL indicated peptides for 72 h, followed by treatments with the noted dose and time of Adriamycin. Determination of viable/live cells was carried out using an MTT assay as described in Section 2. The bar charts in panels A and B represent means of three and two independent experiments, respectively; bars, SE. (**C**) Indicated cells were either pre-incubated with 6 μg/mL indicated peptides for 72 h or not (Control), followed by treatments with Adriamycin as noted. Cells were then processed for immunofluorescence staining for TAT-CARP-1 peptide (red), γH2AX (green), and DAPI (blue) as detailed in Section 2 and Figure S3 (Supplementary Materials). Insets in **C** show enlarged areas of respective photomicrographs to indicate presence of TAT-CARP-1 peptide and γH2AX in respective columns. The insets in the top and middle rows of the merge column show co-localization of TAT-CARP-1 peptide, and γH2AX (magnification: 250×).

We further tested whether and the extent to which transient and ectopic expression of CARP-1 (636–650) peptide would also interfere with the cell-growth inhibition by Adriamycin. For this purpose, we utilized affinity purified His-TAT-HA-tagged EGFP, the His-TAT-HA-tagged CARP-1 (636–650) peptide, and His-TAT-HA-CARP-1 (636–650 scrambled) peptides. The wild-type MDA-MB-468 or HeLa cells were pre-incubated with His-TAT-HA-EGFP or His-TAT-HA-CARP-1 (636–650) proteins, and presence of GFP and CARP-1 (636–650) was determined by immuno-cytochemical staining followed by photography using a confocal microscope as above. As expected, EGFP and CARP-1 (636–650) proteins were predominantly present in the cytoplasmic region (Figure S8A,B, Supplementary Materials). Next, the cells were transduced with EGFP, CARP-1 (636–650), or CARP-1 (636–650, scrambled) proteins and were separately treated with Adriamycin for various times followed by determination of their viabilities with an MTT-based assay as detailed in Section 2. Expression of CARP-1 (636–650) peptide resulted in a moderate, statistically significant increase in viabilities as compared with respective EGFP-expressing counterparts (Figure 8A,B). Next, the MDA-MB-468 cells were firstly pre-incubated with His-TAT-HA-CARP-1 (636–650), His-TAT-HA-CARP-1 (636–650 scrambled) peptides, or not (Control). The cells were then separately treated with Adriamycin, and stained for presence of respective peptide and γH2AX as described in Section 2. Presence of His-TAT-HA-CARP-1 (636–650), but not its scrambled version, resulted in increased presence of γH2AX (top row, Figure 8C), when compared with cells that had either no peptide (Control) or were pre-incubated with His-TAT-HA-CARP-1 (636–650 scrambled) peptide (Figure S9, Supplementary Materials). Data in Figure 8 would suggest that CARP-1 (636–650) peptide likely competes for Adriamycin-induced CARP-1/H2AX interaction that results in reduced growth inhibition, and an overall increase in γH2AX that likely permits DNA repair and cell survival.

## 4. Discussion

Studies from our laboratory and others demonstrated that CARP-1/CCAR1 is a biphasic regulator of cell growth and apoptosis signaling [11]. Although CARP-1 functions as a co-activator of steroid/thyroid nuclear receptors to promote growth [7], and co-activates β-Catenin in colon cancer cells to promote metastasis [8], its transcriptional co-activation of tumor suppressor p53 regulates apoptosis by Adriamycin [7]. Adriamycin action involves pleiotropic effects that include (a) activation of signal transduction pathways, (b) generation of reactive oxygen species (ROS), (c) stimulation of apoptosis, and (d) inhibition of DNA topoisomerase II catalytic activity [39]. Although Adriamycin activates intrinsic apoptotic pathways, and the endogenous apoptosis modulators Bcl-2 and p53 regulate Adriamycin-dependent apoptosis [40,41], a cellular lipid messenger, ceramide, generated by Ariamycin was recently revealed to contribute to the development of resistance in a number of cancer cell types [42]. Nevertheless, inhibition of topoisomerase II by Adriamycin results in the formation of DNA double-strand breaks (DSBs) while formation of intercalating Adriamycin DNA adducts prevent DNA replication [39,40,41]. Overall, DNA damage is key to Adriamycin-induced cell death. Our data from the comet assay revealed, for the first time, that CFM compounds function in part by inducing DNA damage in the cancer cells. Of note is the fact that, although Adriamycin and related anthracyclines function in part by inducing DSBs, it is unclear whether CFM-4.16 also targets cellular DNA and/or the associated DNA repair complex protein(s) to introduce DSBs or activate caspase-activated DNAses to break down cellular genomic DNA.

A number of reports revealed that DNA damage repair and apoptotic signaling by ATM/ATR and JNKs, respectively, involve phosphorylation of H2AX at serine 139 (γ-H2AX). The activation of H2AX by ATM/ATR kinases facilitates DNA repair during low to moderate levels of DNA damage that will likely allow cells to overcome damage and survive. Our findings in Figure 1 show that the H2AX activation was co-incident with increased CARP-1 and activation of pro-apoptotic JNKs. Since DSB-induced apoptosis involves serine 139 phosphorylation of H2AX by JNKs [21], we speculated that CARP-1 was involved in regulating apoptosis by JNK–H2AX signaling. Our co-IP/WB analyses revealed interaction of CARP-1 and H2AX (Figure 3), while knock-down of CARP-1 prevented activation of H2AX by Adriamycin or CFM-4.16 compound. Further mutagenesis studies in Figure 4 and Figure 5 confirmed H2AX–CARP-1 interaction and direct binding, and requirement of their interaction in JNK activation by Adriamycin or CFM-4.16. Additionally, our data demonstrate that CARP-1 amino acids 636–650 and H2AX residues 1–35 harbor minimal epitopes for their respective binding with robust dissociation kinetics.

Our studies presented in Figure 7 demonstrate, for the first time, that abrogation of H2AX–CARP-1 interaction interferes with the inhibitory effects of Adriamycin or CFM compounds, in part through attenuation of apoptosis. More importantly, overexpression of H2AX (1–35) or CARP-1 (636–650) peptides conferred resistance to inhibition by Adriamycin or CFM-4.16. Since these peptides are expected to target the endogenous H2AX–CARP-1 interaction, our “proof-of-concept” studies in Figure 7 demonstrate that H2AX–CARP-1 interaction and consequent signaling could potentially be targeted to attenuate apoptosis signaling. Cardiac and nephro-toxicities associated with many chemotherapeutics including the topoisomerase toxins [33] are well known to prevent optimal utilization in the clinic. If proven, our small peptides or their futuristic small-molecule mimetics could, in principle, be useful means to minimize inhibitory effects of chemotherapy such as Adriamycin in non-cancer, normal tissues of the host.

CARP-1/CCAR1 and its paralog deleted in breast cancer 1 (DBC1, CCAR2, KIAA1967) are large proteins that share many highly conserved protein domains, and have similar patterns of predicted disorder in less-conserved intrinsically disordered regions [43]. DBC1 was previously shown to regulate mitochondrial apoptosis during TNFα-mediated death signaling [44]. Additional reports revealed that DNA damage induced by ionizing radiation or topoisomerase toxin etoposide stimulated DBC1 Thr454 phosphorylation by ATM/ATR kinases. This functional modification of DBC1 promotes binding with deacetylase SIRT1, and consequent SIRT1 inactivation results in p53-dependent apoptotic response [2]. DBC1 was also found to function as a tumor suppressor in part through its ability to regulate p53 stability by competing with MDM2 [45]. In this regard, DBC1 activation of p53 response overlaps with the Adriamycin-induced CARP-1/CCAR1 co-activation function of p53 [7]. Although ATM/ATR kinases regulate DNA damage response (DDR) in part by stimulating γH2AX, and since CARP-1 and DBC1 proteins share many conserved domains, our additional co-immunoprecipitation studies (Figure S10, Supplementary Materials) revealed that DBC1 does not interact with wild-type H2AX or H2AX (1–35) epitope. These findings would suggest that H2AX-mediated apoptosis induced by DNA-damage signaling selectively involves H2AX binding with CCAR1/CARP-1 but not CCAR2/DBC1.

## 5. Conclusions

In conclusion, our studies demonstrate a novel mechanism of apoptosis signaling that is dependent on the unique interaction of CARP-1 and H2AX proteins. Disruption of this interaction could potentially be a useful mechanism to interfere with apoptosis following DNA damage. Our proof-of-concept studies could potentially be extended to cardiomyocytes, and represent a rational means to minimize systemic toxicities, particularly to the cardiac tissues following treatments with DNA-damage-inducing chemotherapies such as Adriamycin. If proven, these mechanism-based, apoptosis-preventing strategies could help optimize anti-cancer efficacy of chemotherapeutics while lowering their systemic/cardiotoxicities.

## Figures and Tables

**Table 1:** Calculation of the binding energies (BE) (Kcal/M) of the CCAR1/H2AX model interactions using MM-PBSA and MM-GBSA.

Approaches	CCAR1/H2AX Model A						CCAR1/H2AX Model B			
	1		2		3		1		2	
	B.E.	Std. Dev.	B.E.	Std. Dev.	B.E.	Std. Dev.	B.E.	Std. Dev.	B.E.	Std. Dev.
MM-PBSA	−31.4	5.39	−21.8	3.97	−37.8	6.80	−24.5	6.00	−44.2	5.46
MM-GBSA	−27.0	6.44	−17.6	3.47	−25.5	8.56	−20.5	5.07	−37.6	6.04

## Supplementary Materials

The following are available online at http://www.mdpi.com/2072-6694/11/2/221/s1: Figure S1: Adriamycin, CFM-4, and CFM-4.16 inhibit cell growth, elevate CARP-1 expression, and induce activation of JNKs and H2AX, and apoptosis, Figure S2: Adriamycin and CFM-4.16 induce DNA damage; Table S1: Adriamycin and CFM-4.16 stimulate DNA Damage in a time-dependent manner, Figure S3: Knock-down of CARP-1 abrogates γH2AX following treatments with Adriamycin or CFM-4.16, Figure S4: Deletion of CARP-1 amino acids 600–650 abrogates activation of H2AX by Adriamycin or CFM-4.16, Figure S5: Characterization of stable sublines expressing EGFP, EGFP-H2AX (1–35), or EGFP-CARP-1 (636–650), Figure S6: Computational Analyses of H2AX (1–35) binding with CARP-1 (631–650), Figure S7: Disruption of H2AX interaction with CARP-1 results in enhanced viabilities of CFM-4, CFM-4.16, or Adriamycin-treated cells in part due to reduced γH2AX and apoptosis, Figure S8: Transduction and cytoplasmic localization of TAT-tagged peptides, Figure S9: Levels of γH2AX in Adriamycin-treated cells harboring His-TAT-HA-CARP-1 (636–650) or His-TAT-HA-CARP-1 (636–650 scrambled) peptides, Figure S10: H2AX does not interact with DBC1 (CCAR2).
