# Supplementary material for: A H2AX–CARP-1 Interaction Regulates Apoptosis Signaling Following DNA Damage
Source: Cancers (Basel). 2019 Feb 14;11(2):221. doi: 10.3390/cancers11020221 (PMC6406907; doi:10.3390/cancers11020221)
Supplement: Supplementary file 1 [file cancers-11-00221-s001.pdf]

Supplementary Materials: A H2AX–CARP-1 Interaction Regulates Apoptosis Signaling Following DNA Damage

Sreeja C. Sekhar, Jaganathan Venkatesh, Vino T. Cheriyan, Magesh Muthu, Edi Levi, Hadeel Assad, Paul Meister, Vishnu V. Undyala, James W. Gauld and Arun K. Rishi

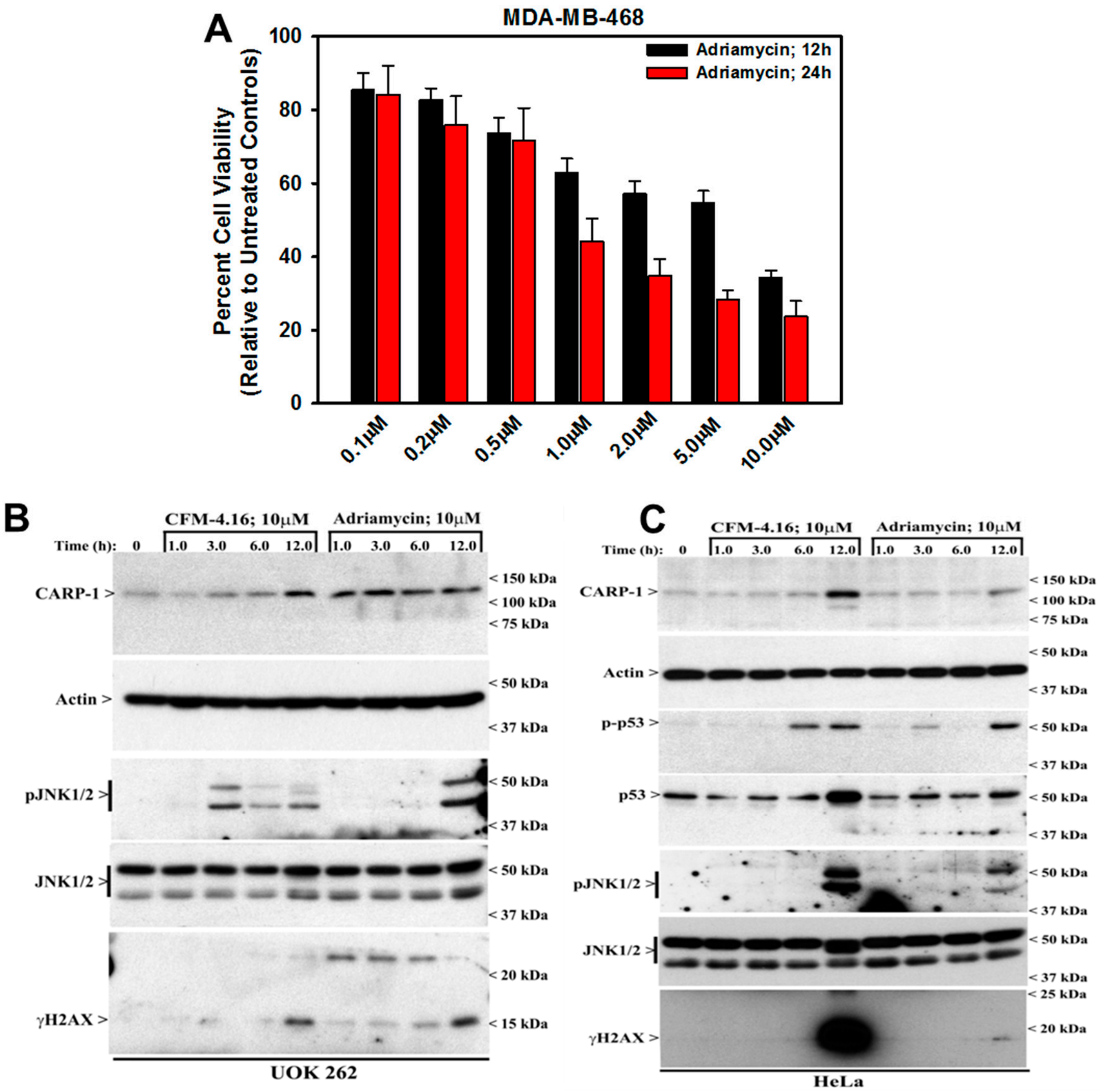

Figure S1. Adriamycin, or CFM-4.16 inhibit cell growth, elevate CARP-1 expression, and induce activation of JNKs and H2AX, and apoptosis. (**A**) MDA-MB-468 cells were either treated with DMSO (Control) or Adriamycin for indicated dose and time. Cell viability was determined by MTT assay. The columns in the bar chart represent means of three independent experiments; bars, SE. The kidney cancer UOK262 (**B**) or HeLa (**C**) cells were either untreated (noted as 0), treated with Adriamycin or CFM-4.16 for noted dose and time. Cell lysates were analyzed by WB as in Methods for levels of CARP-1, phospho- and total JNK1/2, phospho and total p53, and γH2AX. The western blot membranes were subsequently probed with anti-actin antibodies to assess equal loading. The presence of respective protein is indicated by an arrowhead on the left side of each blot. Approximate location of various molecular weight markers is indicated on the right side of each blot. kDa, kilodalton.

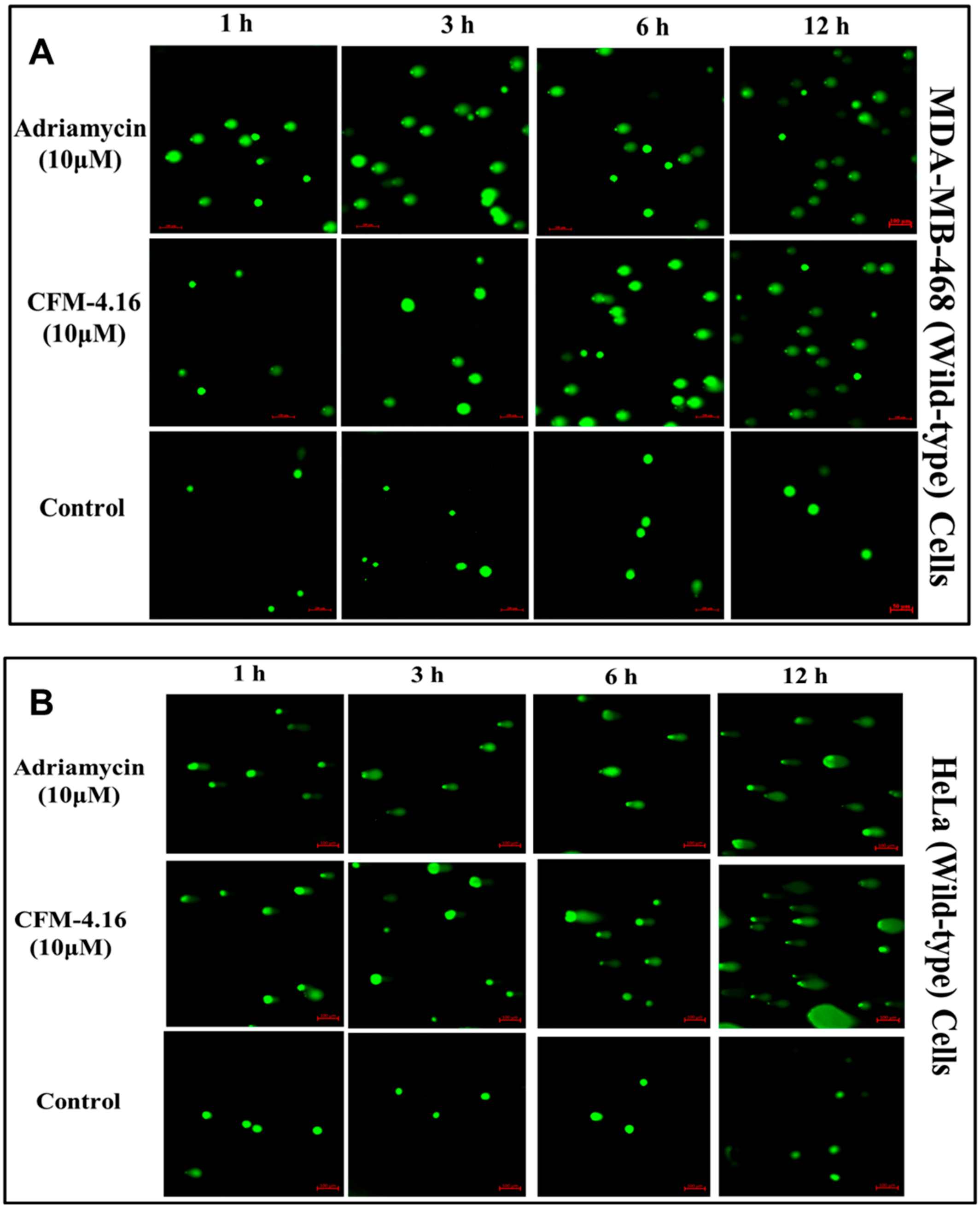

Figure S2. Adriamycin or CFM-4.16 induce DNA damage. Representative photomicrographs showing comet tails of damaged DNA of MDA-MB-468 (**A**) or HeLa (**B**) cells that were either untreated (Control), treated with noted dose of Adriamycin or CFM-4.16 for indicated time periods. Scale bar: 100 µm.

Table S1. Adriamycin and CFM-4.16 stimulate DNA Damage in a time-dependent manner. MDA-MB-468 and HeLa cells were with noted dose of respective compound for indicated time periods. Mean values for % tail DNA, Tail moment, and Olive tail moment for respective cells from control and treatment periods for each compound were measured using software recommended by the manufacturer of the kit for measurement of DNA damage (Cell Bio Labs, San Diego, CA, USA). The *p* values for each of the parameter are indicated below the mean values in each column for the treatment time period with each compound for both the cell lines.

**HeLa Cells**									
		**Adriamycin (10 µM)**				**CFM-4.16 (10 µM)**			
	Control	1 h	3 h	6 h	12 h	1 h	3 h	6 h	12 h
Counted Cell #	87	93	84	89	97	86	92	94	87
%Tail DNA (Mean)	28.414	40.8	76.74	84.98	93.4	27.297	34.45	53.24	69.61
p-value		0.134925	0.000666	0.005966	0.000535	0.004037	0.146319	0.001577	0.003753
Tail Moment (Mean)	9.081	32.09	61.8	79.11	102.2	16.526	26.7	47.54	76.03
p-value		0.001075	7.25E-05	0.020954	0.00274	0.006019	0.07763	0.007131	0.013946
Olive Tail Moment (Mean)	5.539	18.8	39.9	47.9	60.49	10.9	15.4	32.73	41.02
p-value		0.001085	1.15E-05	0.012111	0.001699	0.001162	0.052115	0.004346	0.007842
**MDA-MB-468 Cells**									
		**Adriamycin (10 µM)**				**CFM-4.16 (10 µM)**			
	Control	1 h	3 h	6 h	12 h	1 h	3 h	6 h	12 h
Counted Cell #	94	99	87	86	93	88	99	95	92
%Tail DNA (Mean)	2.39052	18.74701	25.74731	45.54598	63.02	2.507286	17.24032	36.18052	55.6
p-value		0.000773	0.000246	2.07E-07	2.08E-05	0.472458	0.047047	0.000977	0.001203
Tail Moment (Mean)	0.031056	3.462367	14.01461	24.19013	43.07	0.150437	7.449583	25.29162	45.02
p-value		0.001492	6.98E-06	1.34E-09	0.001688	0.223297	0.027359	0.002977	0.030104
Olive Tail Moment (Mean)	0.286711	2.897974	9.946358	14.05645	20.46	0.777259	4.593671	13.06121	24.23
p-value		9.72E-05	4.07E-06	3.87E-08	0.009045	0.426749	0.007602	0.000481	0.007818

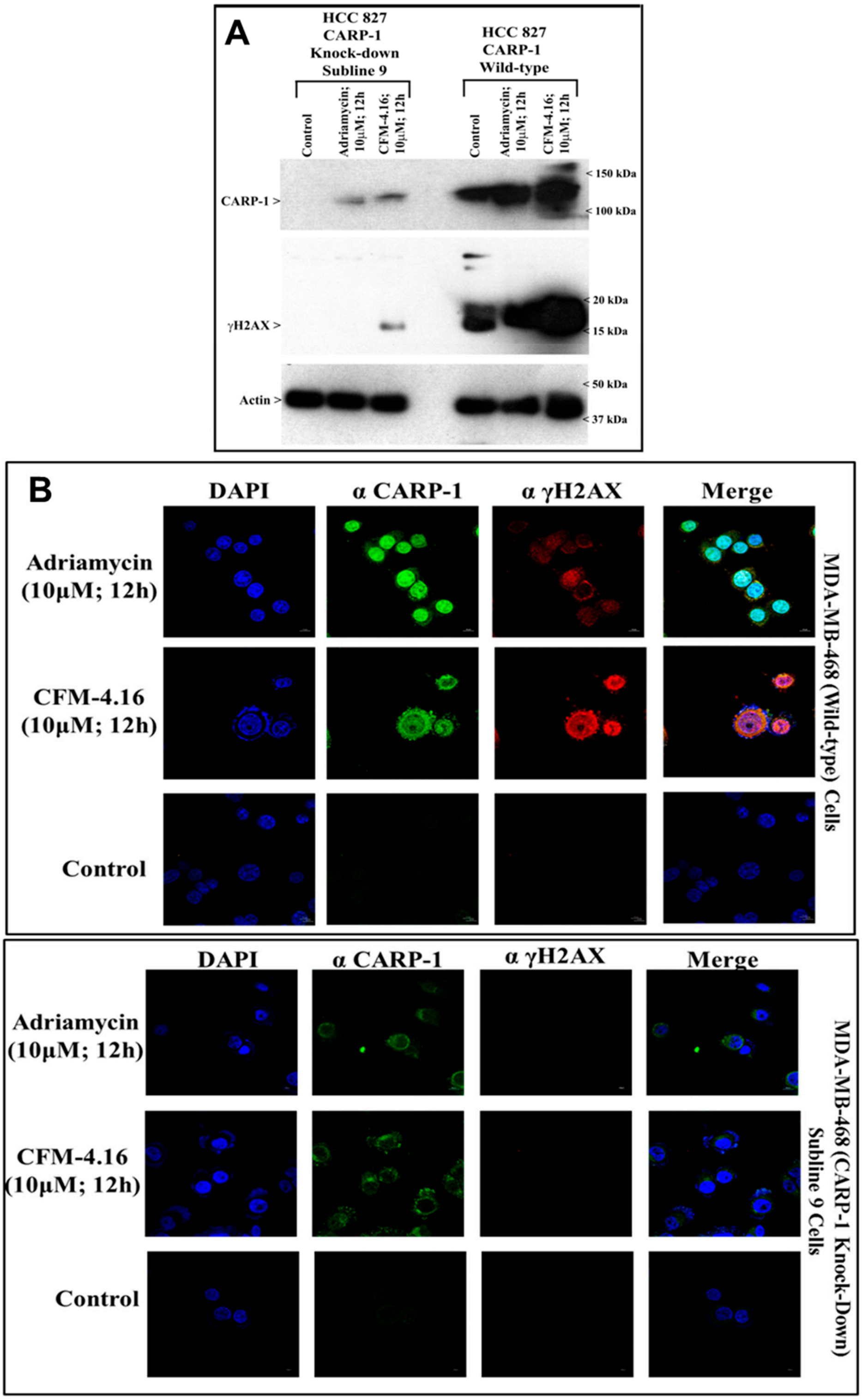

Figure S3. Knock-down of CARP-1 abrogates γH2AX following treatments with Adriamycin or CFM-4.16. (**A**) Cells were either untreated (Control), treated with Adriamycin or CFM-4.16 for noted dose and time. Cell lysates were analyzed by WB as in Methods for levels of CARP-1 and γH2AX. The WB membrane was subsequently probed with anti-actin antibodies to assess equal loading. The presence of respective proteins is indicated by an arrowhead on the left side of each blot. Approximate location of various molecular weight markers is indicated on the right side of each blot. kDa, kilodalton. (**B**) Adriamycin or CFM-4.16 treatments fail to induce CARP-1 or γH2AX in CARP-1-depleted cells. Cells were either untreated (control) or treated with noted time and dose of respective agents. Cells were then processed for immunofluorescence staining for CARP-1 (green), γH2AX (red) and DAPI (blue) as detailed in methods. Images were taken using Zeiss LSM 510 Meta NLO (magnification: 63×).

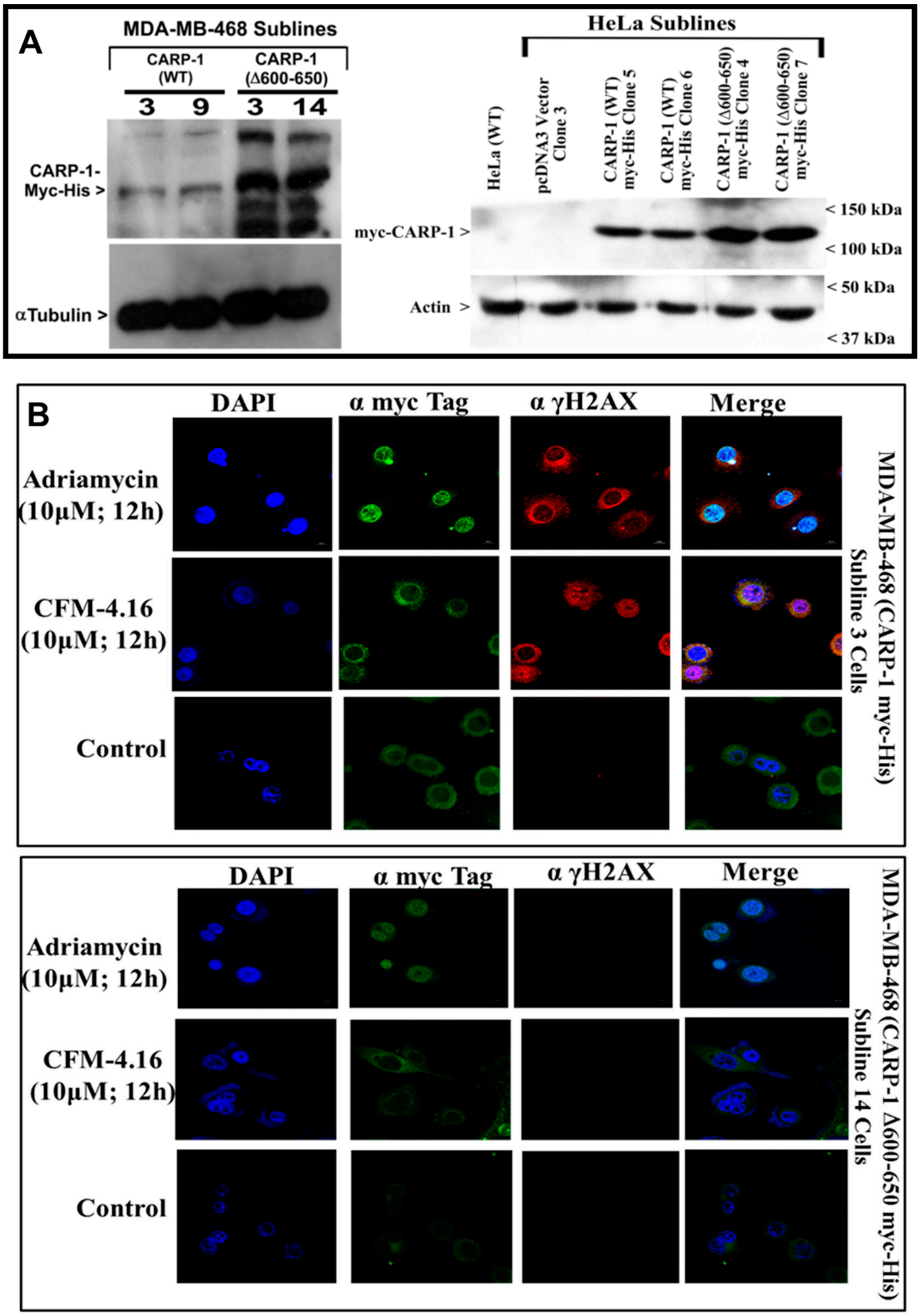

Figure S4. Deletion of CARP-1 amino acids 600–650 abrogates activation of H2AX by Adriamycin or CFM-4.16. (**A**) WB analysis of MDA-MB-468 and HeLa stable sublines expressing myc-His-tagged CARP-1 (WT) or CARP-1 (Δ600–650) proteins. The whole cell lysates were separately analyzed by WB, and the membranes containing proteins were first probed with anti-myc tag antibodies, followed by reprobing of the respective membrane with anti-α-tubulin or Actin antibodies to assess loading. (**B**) Adriamycin or CFM-4.16 treatments fail to induce γH2AX in cell expressing CARP-1 (Δ600–650) mutant. Cells were either untreated (control) or treated with noted time and dose of respective agents. Cells were then processed for immunofluorescence staining for CARP-1 (green), γH2AX (red) and DAPI (blue) as detailed in methods and Figure S2 above. (magnification: 63×)

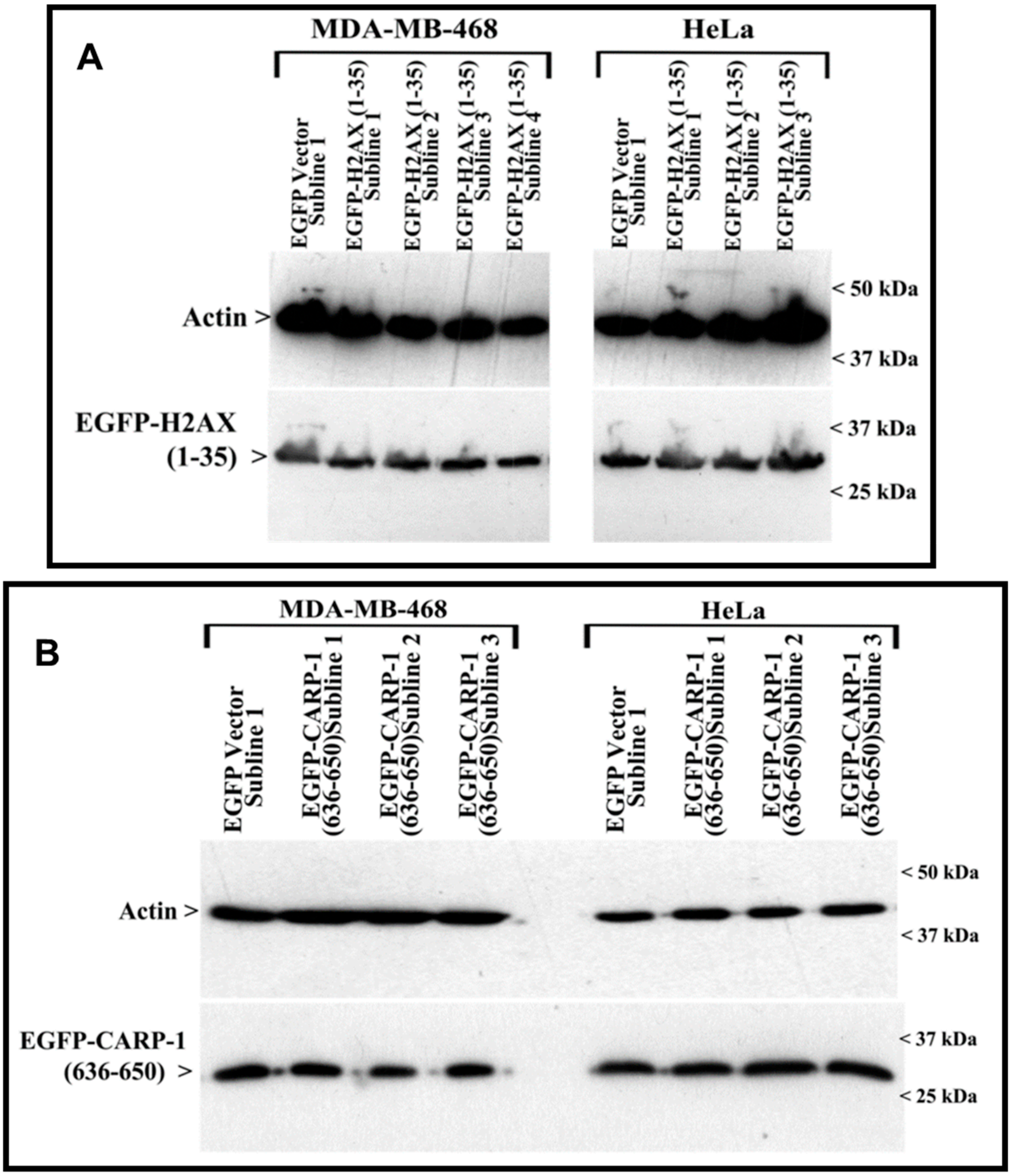

Figure S5. Characterization of stable sublines expressing EGFP, EGFP-H2AX (1–35), or EGFP-CARP-1 (636–650). (**A**,**B**) WB analysis of MDA-MB-468 and HeLa stable sublines expressing EGFP, EGFP-H2AX (1–35) or EGFP-CARP-1 (636–650) proteins. The whole cell lysates were separately analyzed by WB, and the membranes containing proteins were first probed with anti-EGFP antibodies, followed by reprobing of the respective membrane with anti-Actin antibodies to assess loading. The presence of respective proteins is indicated by an arrowhead on the left side of each blot. Approximate location of various molecular weight markers is indicated on the right side of each blot. kDa, kilodalton.

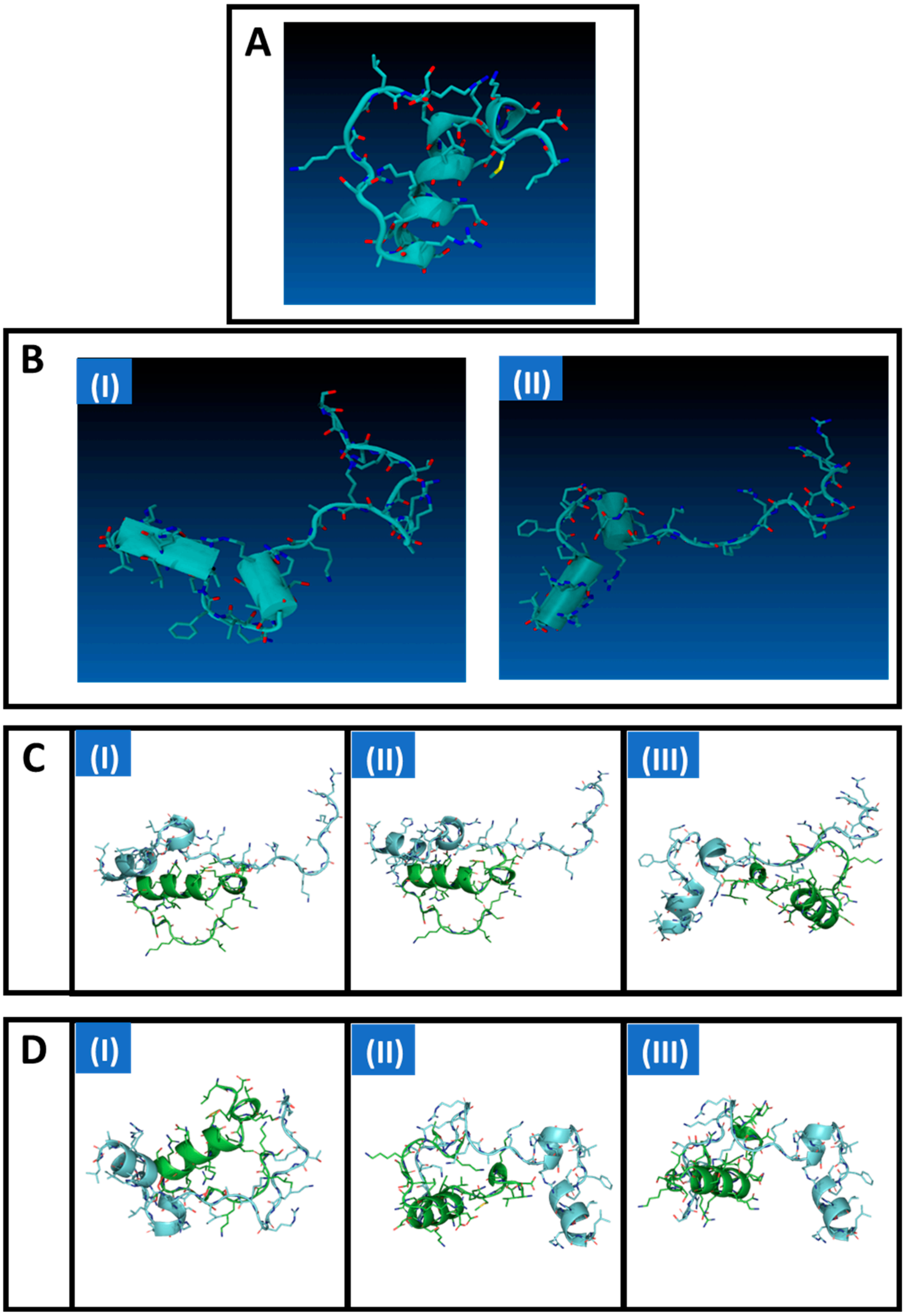

Figure S6. Computational Analyses of H2AX (1–35) binding with CARP-1 (631–650). (**A**) Image of the SWISS-MODEL peptide of the CARP-1 (631–650) epitope that interacts with H2AX (1–35) peptide. (**B**) Images of the SWISS-MODEL peptides of the H2AX (1–35) epitope that interacts with CARP-1 (631–650). Model (I) contains an extra N-terminal serine compared to model (II). (**C**) Top three scoring CARP-1 (631–650)/H2AX (1–35) docked complexes obtained using Model A (see BI above) for H2AX, in descending order: (I), (II), then (III). (**D**) The top three CARP-1 (631–650)/H2AX (1–35) docked complexes obtained using model B (see BII above) for H2AX, in descending order: (I), (II), then (III). H2AX peptide is in light blue while CARP-1 peptide is in green color.

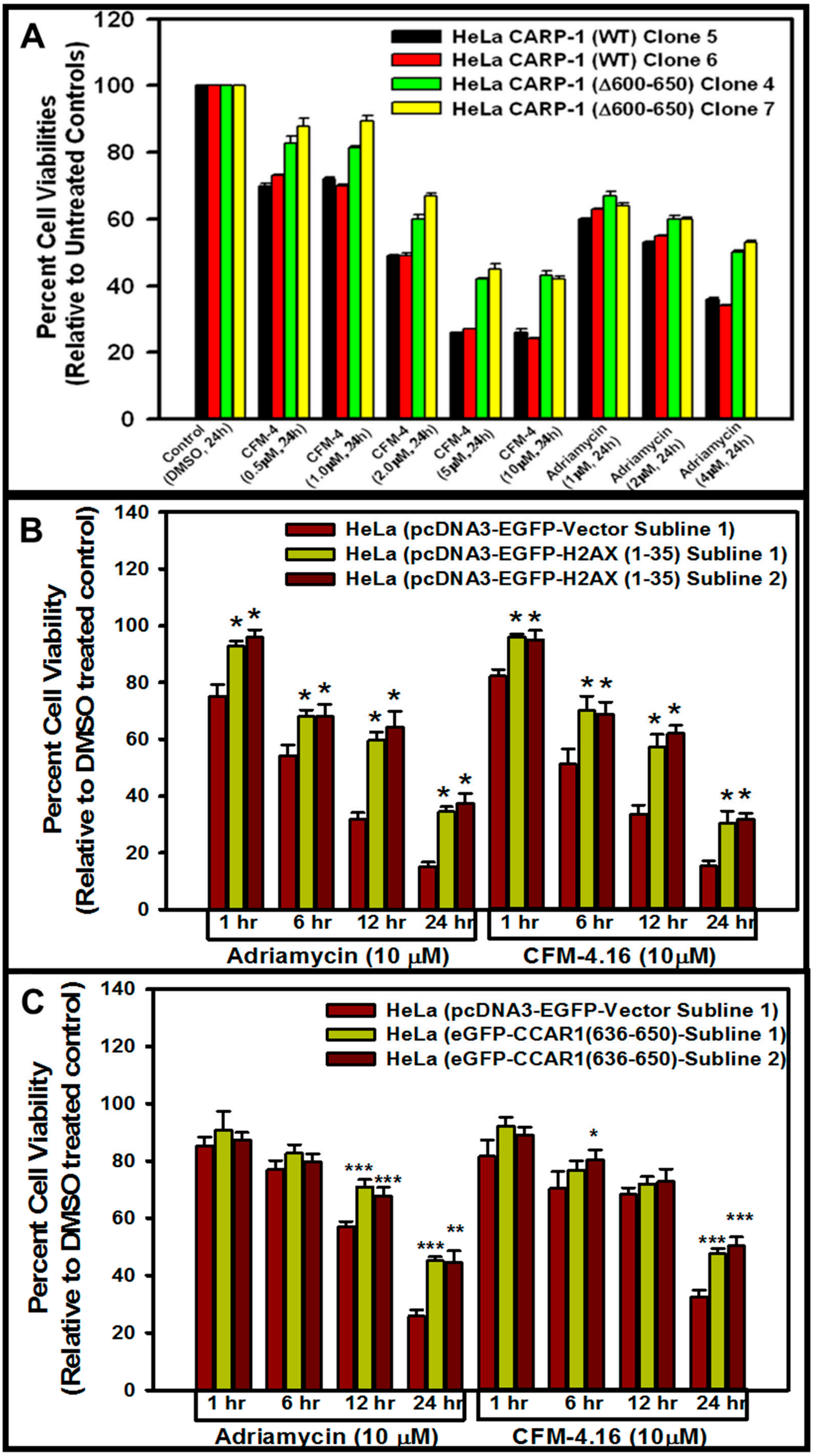
A
Percent Cell Viabilities (Relative to Untreated Controls)
HeLa CARP-1 (WT) Clone 5
HeLa CARP-1 (WT) Clone 6
HeLa CARP-1 (Δ600-650) Clone 4
HeLa CARP-1 (Δ600-650) Clone 7
Control (DMSO, 24h)
CFM-4 (0.5μM, 24h)
CFM-4 (1.0μM, 24h)
CFM-4 (2.0μM, 24h)
CFM-4 (5μM, 24h)
CFM-4 (10μM, 24h)
Adriamycin (1μM, 24h)
Adriamycin (2μM, 24h)
Adriamycin (4μM, 24h)
B
Percent Cell Viability (Relative to DMSO treated control)
HeLa (pcDNA3-EGFP-Vector Subline 1)
HeLa (pcDNA3-EGFP-H2AX (1-35) Subline 1)
HeLa (pcDNA3-EGFP-H2AX (1-35) Subline 2)
1 hr 6 hr 12 hr 24 hr
Adriamycin (10 μM)
CFM-4.16 (10μM)
C
Percent Cell Viability (Relative to DMSO treated control)
HeLa (pcDNA3-EGFP-Vector Subline 1)
HeLa (eGFP-CCAR1(636-650)-Subline 1)
HeLa (eGFP-CCAR1(636-650)-Subline 2)
1 hr 6 hr 12 hr 24 hr
Adriamycin (10 μM)
CFM-4.16 (10μM)

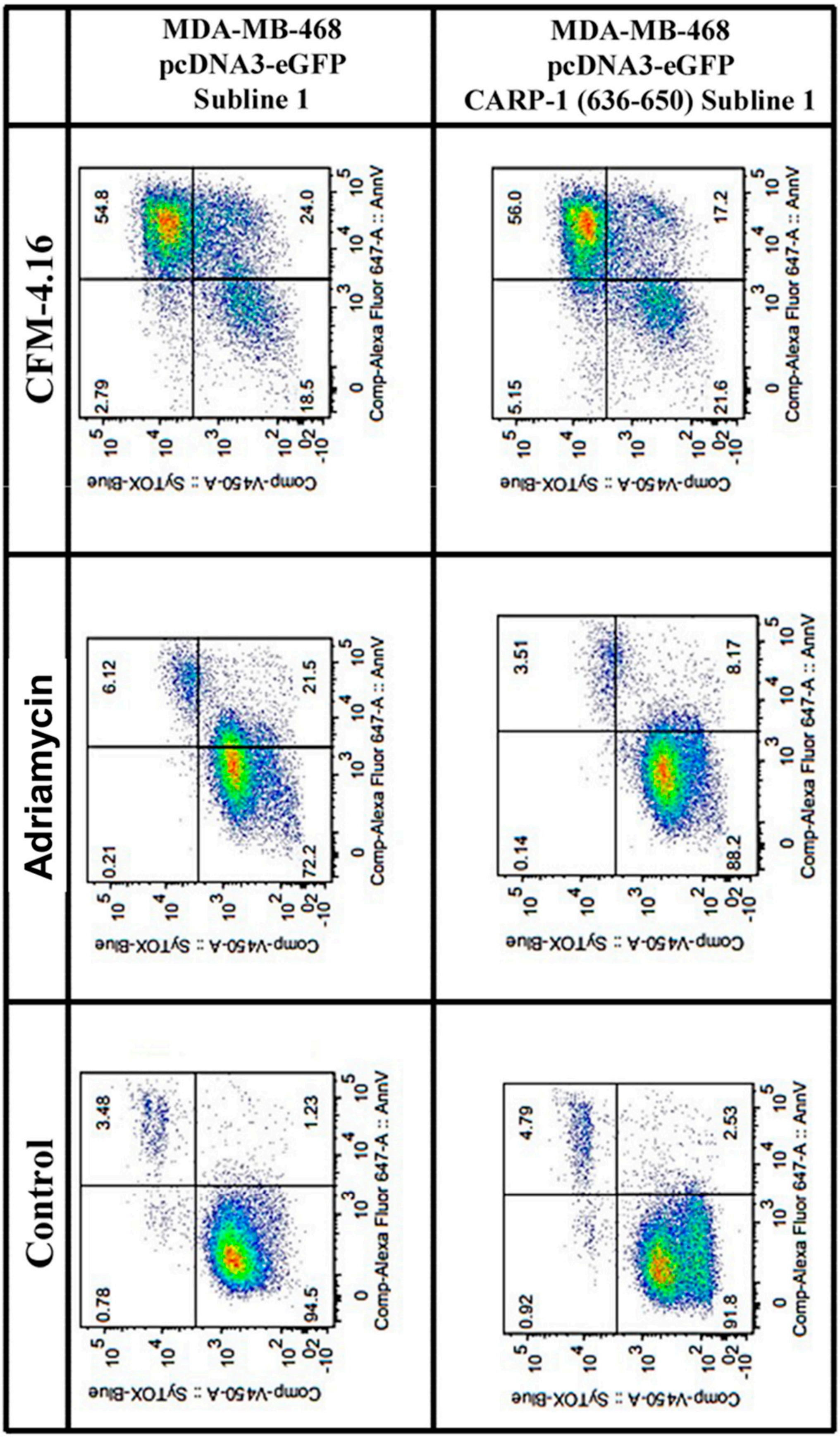

Supplementary Figure 7D

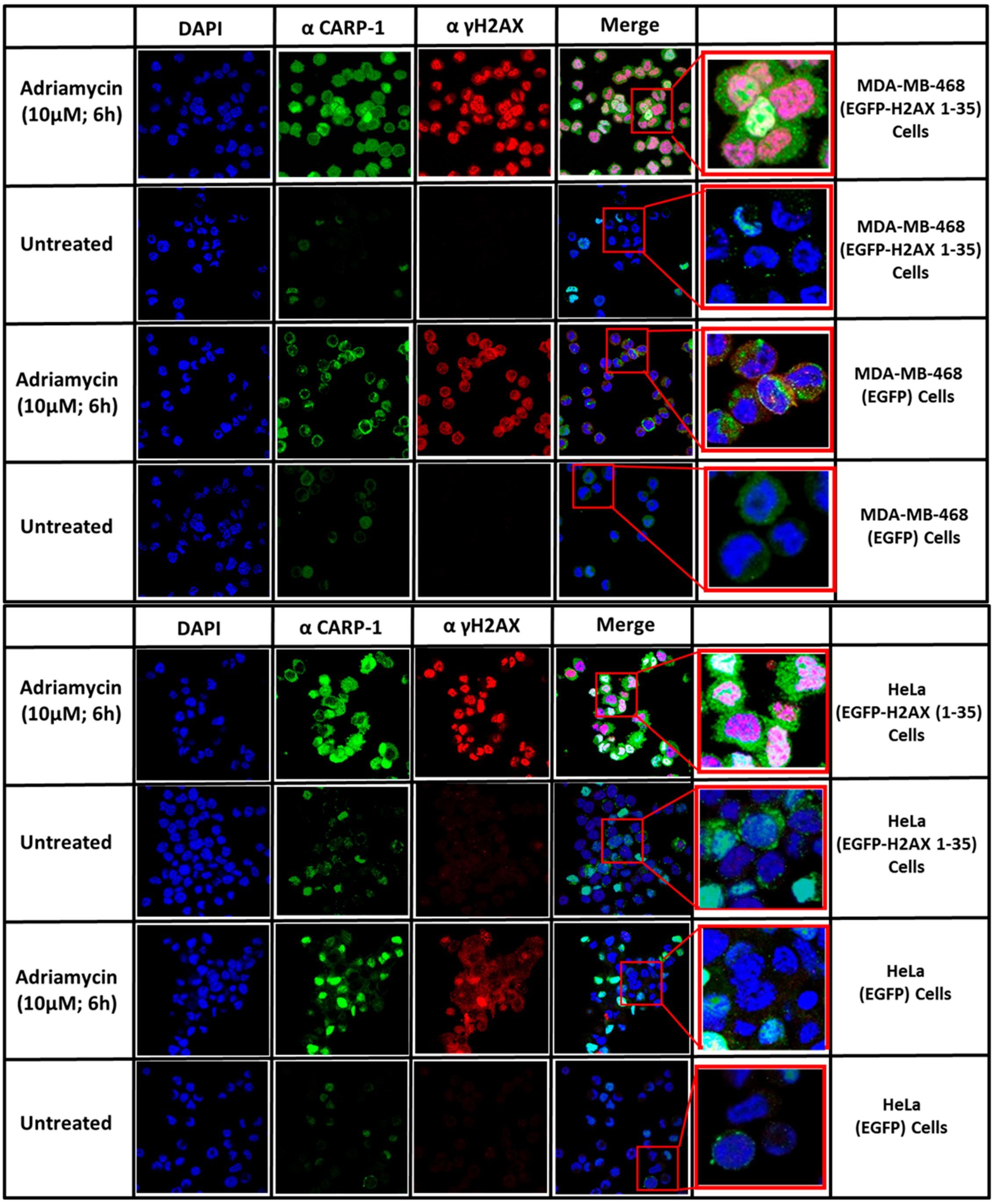

Supplementary Figure 7E

Figure S7. Disruption of H2AX interaction with CARP-1 results in enhanced viabilities of CFM-4, CFM-4.16, or Adriamycin-treated cells in part due to reduced γH2AX and apoptosis. (**A**,**B**,**C**) Indicated HeLa cell lines were treated with DMSO (Control), noted dose and time of Adriamycin, CFM-4, or CFM-4.16 compounds. Determination of viable/live cells was carried out by MTT assay as in methods. The columns in bar charts in panels A, B, C represent means of three independent experiments; bars, SE. For panels B and C, * $p < 0.05$, ** $p < 0.01$ and ***, $p \leq 0.001$ relative to the respective EGFP Vector subline. (**D**) Flow cytometric analysis of MDA-MB-468 stable sublines expressing EGFP or EGFP-CARP-1 (636–650) that were either untreated (Control), treated for 24 h with 10 μM dose of Adriamycin or CFM-4.16. The cell #s are indicated in each quadrant. (**E**) Adriamycin treatments induce nuclear localization of γH2AX in cell expressing EGFP-H2AX (1–35) peptide. Cells were either untreated (control) or treated with noted time and dose of Adriamycin. Cells were then processed for immunofluorescence staining for CARP-1 (green), γH2AX (red) and DAPI (blue) as detailed in methods and Figure S3 above. (magnification: 63×; 250× for insets).

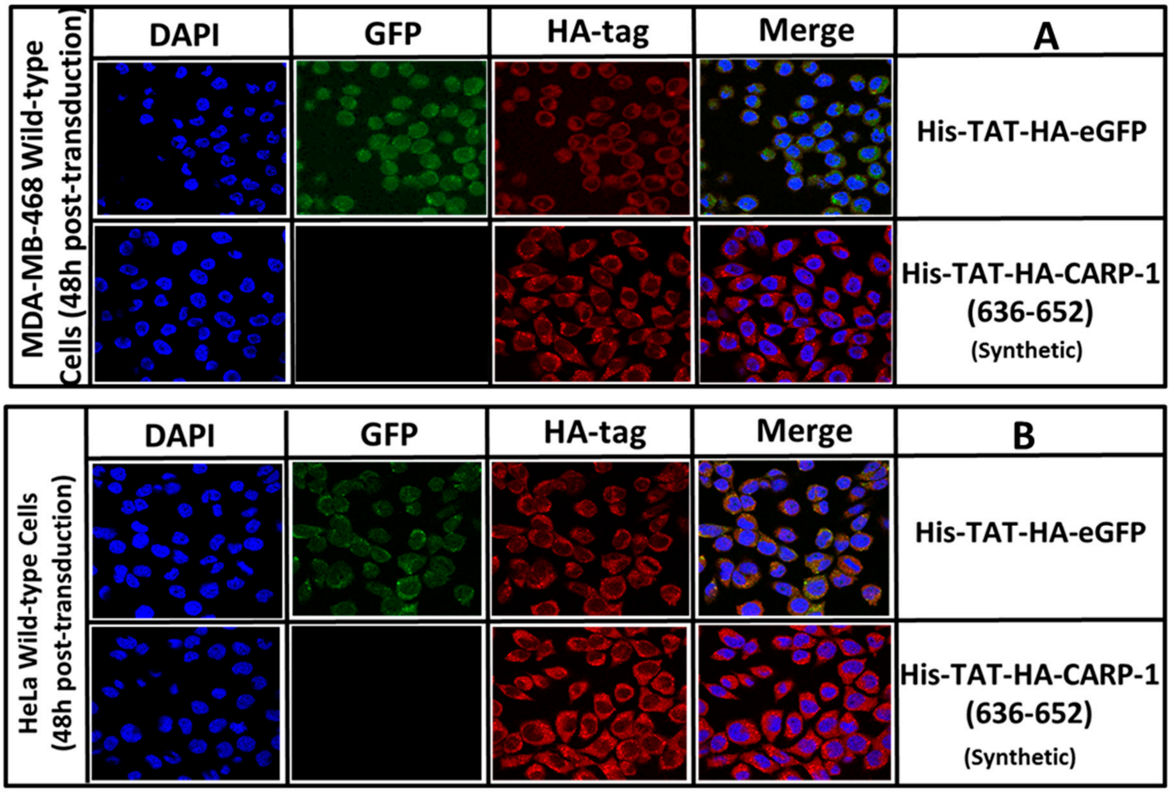

Figure S8. Transduction and cytoplasmic localization of TAT-tagged peptides. MDA-MB-468 (**A**) or HeLa (**B**) cells were pre-incubated with 300 µg/mL of indicated peptide for noted time. Cells were then processed for immunofluorescence staining for TAT-tagged peptides (red), GFP (green) and DAPI (blue) as detailed in methods and Figure S2 above. (magnification: 63×).

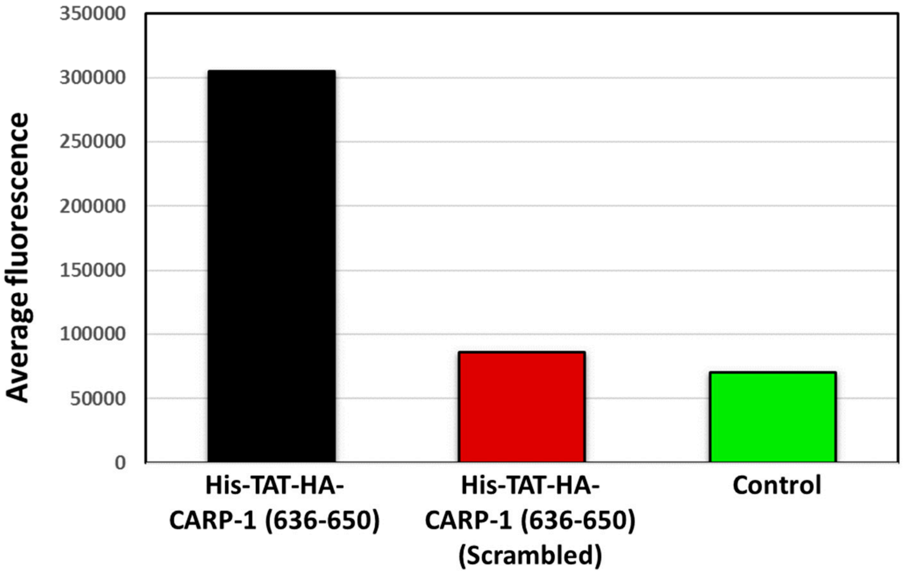

Figure S9. Levels of γH2AX in Adriamycin-treated cells harboring His-TAT-HA-CARP-1 (636–650) or His-TAT-HA-CARP-1 (636–650 scrambled) peptides. Multiple cells from each of the confocal images in the H2AX column of Figure 8C were subjected to quantitation for levels of H2AX. The quantitation of the images of stained cells was carried out by using ImageJ software (imagej.nih.gov/ij/plugins/track/jacop2). The columns in the bar chart represent average fluorescence from H2AX-stained cells in five random cells of each of the respective row.

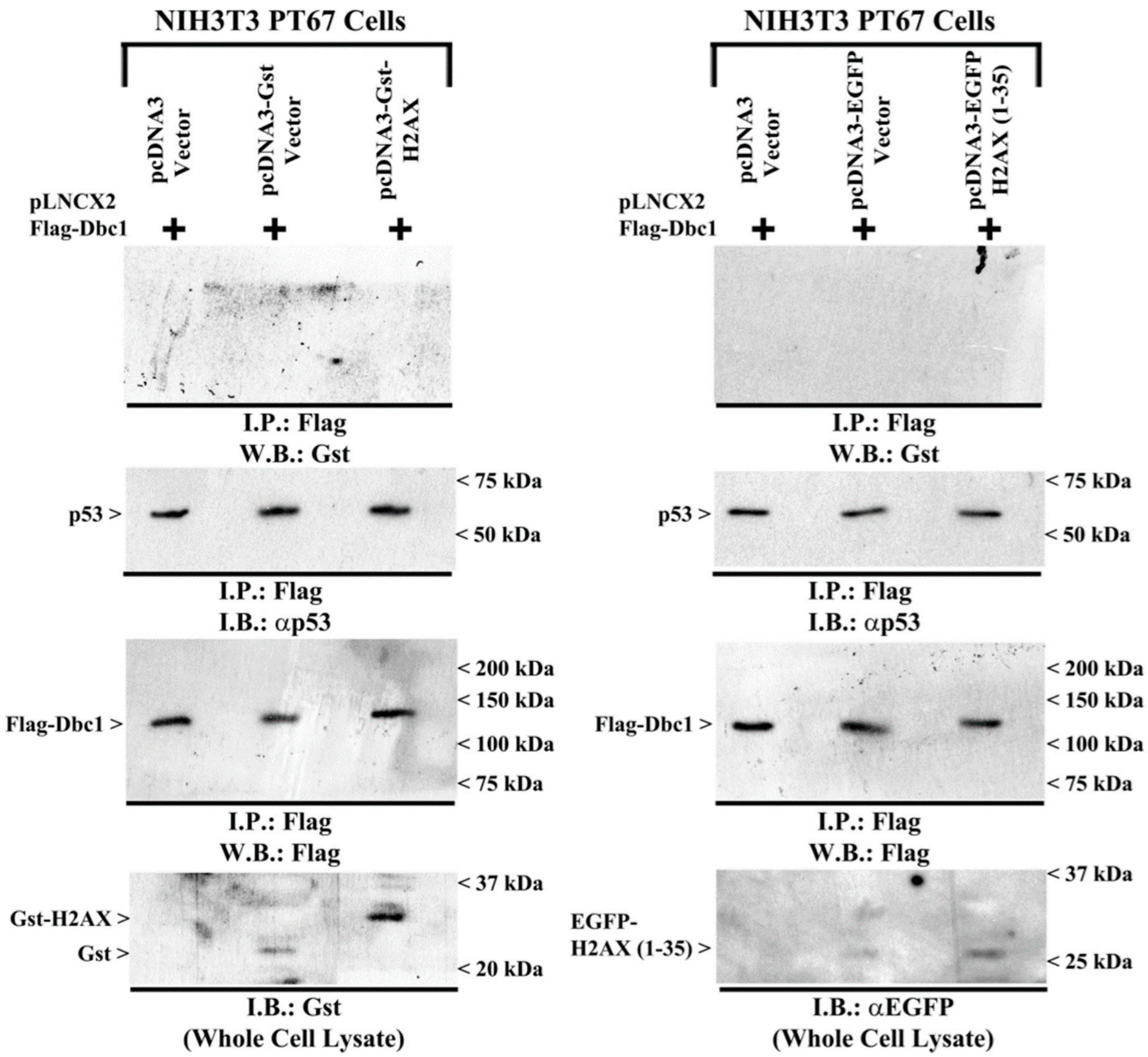

Figure S10. H2AX does not interact with Dbc1 (CCAR2): NIH3T3 murine fibroblast subline PT67 (Clontech) were first transfected with recombinant PLNCX2/Flag-Dbc1 plasmid (10), and stable, neomycin-resistant sublines were generated and characterized for Dbc1 expression essentially as described by us before (5; [35]). The Dbc1 expressing PT67 cells were then separately transfected with pcDNA3 vector, pcDNA3-Gst, pcDNA3-Gst-H2AX, pcDNA3-EGFP, or pcDNA3-EGFP-H2AX (1–35) plasmids. Approximately 1mg of cell lysate from each of transfected, Dbc1-expressing, PT67 cells was subjected to immunoprecipitation using anti-Flag tag M2 antibodies (F1804, Sigma). The immunoprecipitates were then analyzed by WB by probing the membrane with anti-Gst (top autoradiograph), anti-p53 D2H90 rodent specific rabbit mAb (32532S; Cell Signaling; 2nd autoradiograph from top), or anti-Fag tag (for Dbc1) antibodies (3rd autoradiograph from top). The membranes containing 50 mg of respective cell lysates (marked as whole cell lysates) were subsequently probed with anti-Gst tag (Bottom left autoradiograph) or anti-EGFP antibodies (Bottom right autoradiograph) to ascertain expression of transfected, Gst, Gst-tagged H2AX proteins, EGFP, and EGFP-H2AX (1–35) proteins. The presence of respective proteins is indicated by an arrowhead on the left side of each blot. Approximate location of various molecular weight markers is indicated on the right side of each blot. kDa, kilodalton.

© 2019 by the authors. Licensee MDPI, Basel, Switzerland. This article is an open access article distributed under the terms and conditions of the Creative Commons Attribution (CC BY) license (http://creativecommons.org/licenses/by/4.0/).
